# STAT1 is a sex‐specific tumor suppressor in colitis‐associated colorectal cancer

**DOI:** 10.1002/1878-0261.12178

**Published:** 2018-02-20

**Authors:** Ilija Crnčec, Madhura Modak, Claire Gordziel, Jasmin Svinka, Irene Scharf, Stefan Moritsch, Paulina Pathria, Michaela Schlederer, Lukas Kenner, Gerald Timelthaler, Mathias Müller, Birgit Strobl, Emilio Casanova, Editha Bayer, Thomas Mohr, Johannes Stöckl, Karlheinz Friedrich, Robert Eferl

**Affiliations:** ^1^ Institute of Cancer Research Medical University Vienna & Comprehensive Cancer Center (CCC) Vienna Austria; ^2^ Institute of Immunology Medical University Vienna Austria; ^3^ Institute of Biochemistry II University Hospital Jena Germany; ^4^ Ludwig Boltzmann Institute for Cancer Research LBICR Vienna Austria; ^5^ Department of Experimental Pathology and Laboratory Animal Pathology Clinical Institute of Pathology Medical University Vienna Austria; ^6^ Unit of Laboratory Animal Pathology University of Veterinary Medicine Vienna Austria; ^7^ Institute of Animal Breeding and Genetics and Biomodels Austria University of Veterinary Medicine Vienna Austria; ^8^ Department of Physiology Center of Physiology and Pharmacology Medical University Vienna Austria

**Keywords:** CD8^+^ T cells, colitis, colorectal cancer, gender, sex, STAT1

## Abstract

The interferon‐inducible transcription factor STAT1 is a tumor suppressor in various malignancies. We investigated sex‐specific STAT1 functions in colitis and colitis‐associated colorectal cancer (CRC) using mice with specific STAT1 deletion in intestinal epithelial cells (STAT1^∆IEC^). Male but not female STAT1^∆IEC^ mice were more resistant to DSS‐induced colitis than sex‐matched STAT1^flox/flox^ controls and displayed reduced intraepithelial infiltration of CD8^+^ TCRαβ^+^ granzyme B^+^ T cells. Moreover, DSS treatment failed to induce expression of T‐cell‐attracting chemokines in intestinal epithelial cells of male but not of female STAT1^∆IEC^ mice. Application of the AOM‐DSS protocol for induction of colitis‐associated CRC resulted in increased intestinal tumor load in male but not in female STAT1^∆IEC^ mice. A sex‐specific stratification of human CRC patients corroborated the data obtained in mice and revealed that reduced tumor cell‐intrinsic nuclear STAT1 protein expression is a poor prognostic factor in men but not in women. These data demonstrate that epithelial STAT1 is a male‐specific tumor suppressor in CRC of mice and humans.

**Abbreviations**
AOM: azoxymethane
CMS: consensus molecular subtype
CRC: colorectal cancer
CXCL: chemokine (C‐X‐C motif) ligand
DSS: dextran sodium sulfate
IBD: inflammatory bowel disease
IEC: intestinal epithelial cell
IFN: interferon
IHC: immunohistochemistry
IL‐6: interleukin 6
JAK: janus kinase
MACS: magnetic‐activated cell sorting
qRT‐PCR: quantitative reverse transcriptase polymerase chain reaction
SOCS: suppressor of cytokine signaling
STAT: signal transducer and activator of transcription
TCR: T‐cell receptor

## 1 Introduction

The incidence of many cancers is significantly higher in men than in women with only a few exceptions. This sex‐specific susceptibility is due to occupational and behavioral factors, sex‐related hormone signaling, and cancer‐modulating genes on sex chromosomes (Clocchiatti *et al*., 2016). Moreover, immune responses and cancer immune surveillance are sex‐dependent with women mounting stronger innate and adaptive immune responses than men do (Klein and Flanagan, 2016). CRC morbidity is similar in men and women but distinct sex‐specific differences are apparent. Female CRC patients over 65 years show a lower 5‐year survival rate than age‐matched male patients because they develop mainly right‐sided CRC in the proximal colon, which is more aggressive than left‐sided CRC predominating in men. Due to the flat appearance, right‐sided CRC is also more difficult to detect by colonoscopy than polypoid‐like left‐sided CRC. Despite these differences and an ongoing discussion on the clinical implementation of precision medicine, CRC guidelines for screening or therapy do not apply sex‐specific recommendations. This might be due to the limited availability of sex‐specific preclinical data as most animal studies avoided possible implications of estrogen signaling and used male mice for CRC induction (Kim *et al*., 2015). Therefore, it is important to identify sex‐related biological factors that affect CRC formation in men and women differently. These factors might be valuable therapy targets and markers for cancer prognosis after sex stratification of patients.

Colitis patients suffering from inflammatory bowel diseases (IBD) have an increased risk for development of CRC (Terzic *et al*., 2010). Signal transducer and activator of transcription 1 (STAT1) is activated by interferon (IFN) signaling via tyrosine phosphorylation (Murray, 2007; Strobl *et al*., 2011) and was found upregulated in the colon mucosa of IBD patients (Schreiber *et al*., 2002). Murine studies have shown that STAT1 promotes colitis. DSS‐induced colitis was reduced in STAT1 knockout mice (Bandyopadhyay *et al*., 2008; Berry *et al*., 2012) whereas mice with haploinsufficiency of SOCS1, a negative regulator of STAT1, showed more severe colitis (Horino *et al*., 2008). However, a recent study has suggested that epithelial STAT1 protects from DSS‐induced colitis in mice (Chiriac *et al*., 2017).

STAT1 is commonly considered as a tumor suppressor (Meissl *et al*., 2015) as observed in murine sarcomas (Shankaran *et al*., 2001) and several mouse breast cancer models (Chan *et al*., 2012; Klover *et al*., 2010; Raven *et al*., 2011; Schneckenleithner *et al*., 2011). STAT1 inhibits proliferation and promotes apoptosis of tumor cells (Meissl *et al*., 2015). Moreover, STAT1 promotes extrinsic, tumor‐suppressive effects by enhancement of tumor immune surveillance through NK‐ and T cells (Meissl *et al*., 2015). However, the role of STAT1 in CRC is controversial and data for colitis‐associated CRC are limited. An oncogenic function was suggested in SOCS1 knockout mice, which developed sporadic CRC with strong tumor cell‐intrinsic STAT1 activation (Hanada *et al*., 2006), but otherwise, Apc^Min^‐induced intestinal tumorigenesis was not affected in STAT1 knockout mice (Liddle and Frank, 2008). STAT1 functions in CRC might be concealed by dual effects in tumor cells and stromal immune cells, which are both affected in STAT1 knockout mice. Conditional approaches are required to discriminate between these cellular compartments. We employed mice with specific deletion of STAT1 in intestinal epithelial cells to investigate tumor cell‐intrinsic functions in colitis‐associated CRC. We demonstrate a sex‐specific and tumor‐suppressive function of STAT1 in CRC of humans and mice.

## 2 Materials and methods

### 2.1 Mice and *in vivo* experiments

C57BL/6 STAT1^∆IEC^ (Villin^Cre/+^ STAT1^flox/flox^) and control STAT1^flox/flox^ mice (el Marjou *et al*., 2004; Wallner *et al*., 2012) were employed for tumor and colitis induction. Only littermates were used. They were cohoused until weaning age and then separated for experiments. For tumor induction, mice were treated with 12.5 mg·kg^−1^ of AOM (Sigma, A5486) i.p. and subjected after a 5‐day recovery period to two 5‐day cycles of 2.5% DSS (MP Biomedicals, LLC 160110) and one 5‐day cycle of 2% DSS (w/v) in drinking water with a 14‐day interval of normal water between the cycles (Crncec *et al*., 2015). For induction of colitis, mice were given 2.5% DSS (w/v) in drinking water for 7 days followed by a 3‐day recovery period. All mouse experiments were performed in accordance with Austrian and European laws and with the general regulations specified by the Good Science Practices guidelines of the Medical University of Vienna.

### 2.2 Histology and immunohistochemistry (IHC)

Intestines were flushed with PBS and 4% buffered formaldehyde and fixed as Swiss rolls in 4% formaldehyde (Crncec *et al*., 2015). Paraffin sections of 4 μm thickness were stained with H&E, alcian blue or IHC using standard procedures.

### 2.3 Isolation of intraepithelial and lamina propria lymphocytes

Intraepithelial and lamina propria lymphocytes were isolated as described (Weigmann *et al*., 2007). In brief, colons of three mice per genotype and sex were flushed with PBS and opened longitudinally. Large colon pieces were then shaken in a CMF/HEPES solution containing 1 × HBSS, FCS, 10 mm HEPES, and 25 mm sodium bicarbonate 3 × 15 s to remove excess mucus, fecal material, and loose fat. Subsequently, they were washed in RPMI 1640 (Sigma‐Aldrich #R8755) containing FCS. Large pieces were then sliced into 1‐ to 3‐mm pieces and shaken in Hank′s balanced salt solution containing 10% FCS, 5 mm EDTA, 15 mm HEPES and penicillin/streptomycin [100U·mL^−1^] at 37 °C for 2 × 15 min. The supernatant of this preparation was used for isolation of intraepithelial lymphocytes. For lamina propria lymphocytes, the remaining pieces of colon were shaken in RPMI 1640 containing 5% FCS, 1 mm of MgCl_2_, 1 mm CaCl_2_ and Collagenase [100U·mL^−1^] at 37 °C for 2 × 20 min. The supernatant of both preparations was subjected to Percoll gradient centrifugation. The lymphocytes were collected at the interface between 44% and 67% layers for intraepithelial lymphocytes and 40% and 80% layers for lamina propria lymphocytes.

### 2.4 Isolation of intestinal epithelial cells and MACS purification

Intestinal epithelial cells were isolated by shaking 1‐ to 3‐mm colon pieces in Hank′s balanced salt solution containing 10% FCS, 5 mm EDTA, 15 mm HEPES, and penicillin/streptomycin [100U·mL^−1^] at 37 °C for 2 × 15 min. The IECs of the DSS‐treated mice were subjected to MACS purification using BD IMag™ Streptavidin Particles Plus—DM system (BD Biosciences #557812) and biotinylated rat anti‐mouse CD45 (clone 30‐F11; BD Biosciences #553077) according to the manufacturer′s instructions. Isolated IECs were tested by flow cytometry and showed 85–95% purity.

### 2.5 Antibodies used for IHC stainings

IHC stainings were performed with antibodies for STAT1 (Santa Cruz #sc‐592), phospho‐STAT1 (Tyr701) (Cell Signaling #9167S), Ki67 (Novocastra #NCL‐KI67‐P), cleaved caspase‐3 (Cell signaling #9661), STAT3 (Santa Cruz #sc‐7179), phospho‐STAT3 (Tyr705) (Cell signaling #9145), lysozyme (Dako #A0099), synaptophysin (Genetex GTX100865), endomucin (eBioscience #14‐5851‐82), granzyme B (Abcam #ab4059), p21 (Santa Cruz #sc‐6246), c‐Myc (Santa Cruz #sc‐40), IL‐6 (Abcam #ab6672) and detected with peroxidase‐coupled secondary antibody (ID laboratories IDSTM003) using AEC chromogen (ID laboratories #BP1108) or AEC staining kit (Sigma‐Aldrich #AEC 101‐1KT). BrdU incorporation was detected using the BrdU In‐Situ Detection Kit (BD Biosciences #550803).

### 2.6 Quantitation and grading of tumors

Pannoramic MIDI scanner (3DHistech Ltd.) was used to scan H&E‐stained and IHC‐stained Swiss rolls. Quantitation of tumor area and IHC stainings was performed using Definiens™ Tissue Studio histomorphometry software (Definiens AG, Munich, Germany). Tumor grading was performed by a board‐certified pathologist.

### 2.7 Colitis score

Scanned H&E Swiss rolls of colons were used to assess colitis in a blinded fashion by a board‐certified pathologist according to the following criteria: inflammation score 0—rare or no inflammatory cells in lamina propria, 1—increased numbers of granulocytes in lamina propria, 2—confluence of inflammatory cells extending to submucosa, 3—transmural extension of inflammatory infiltrate; crypt damage 0—none, 1—loss of basal 1/3 of the crypt, 2—loss of basal 2/3 of the crypt, 3—entire crypt loss, 4—change in epithelial surface with erosion, 5—confluent erosion; ulceration 0—none, 1—1–2 ulcers focally, 2—3–4 ulcers focally, 3—confluent ulceration. The individual scores were added up to give a maximum of 11. This score was then multiplied by a multiplication factor on the basis of the area affected: 1—0–25% of the colon, 2—25–50% of the colon, 3—50–75% of the colon, 4—75–100% of the colon to give a final maximum score of 44.

### 2.8 Flow cytometry

Flow cytometry analyses were performed using standard staining procedures. For membrane staining, cells were incubated with conjugated mAbs for 30 min at 4 °C. For intracellular staining, cells were fixed with fixation buffer in the dark at 4 °C for 20 min. Cells were then incubated with conjugated mAb in permeabilization buffer at 4 °C for 1 h. Flow cytometry analyses were performed using LSRFortessa (Becton Dickinson, Heidelberg, Germany). Following reagents were used: Fixable Viability Dye eFluor506® (eBioscience #65‐0866‐14) and antibodies CD16/32 (BD Biosciences #553142; clone 2.4G2), CD45–BV421 (Biolegend 103133; clone 30‐F11), CD45–APC‐R700 (BD Biosciences #565478; clone 30‐F11), CD8α ‐ APC (eBioscience #17‐0081‐83; clone 53‐6.7), CD8α ‐ APC‐R700 (BD Biosciences #564983; clone 53‐6.7), CD8α ‐ BV605 (Biolegend #100744; clone 53‐6.7), CD4–PE‐Cy.7 (eBioscience #25‐0042‐82; clone RM4‐5), CD19 ‐ PE‐CF594 (BD Biosciences #562329; clone 1D3), TCRß ‐ APC‐eFluor® 780 (eBioscience #47‐5961‐82; clone H57‐597), TCRγδ ‐ PE (BD Biosciences #553178; clone GL3), granzyme B ‐ PE (BD Biosciences #561142; clone GB11), isotype (BD Biosciences #556650; clone MOPC‐21), CD326 ‐ PE (BD Biosciences #563477; clone G8.8), fixation/permeabilization diluent (eBioscience #00‐5223‐56), permeabilization buffer (10x) (eBioscience #00‐8333‐56), fixation/permeabilization concentrate (eBioscience #00‐5123‐43).

### 2.9 Genotyping by polymerase chain reaction (PCR)

Genotyping of STAT1 was performed with primers 5′‐TAGGCTCCCTCTTTCCCTTC‐3′, 5′‐ACACCATTGGCTTCACCTTC‐3′, and 5′‐CCCCTGTCATCTGGAGTGAT‐3′. The Cre transgene was detected with primers 5′‐CGGTCGATGCAACGAGTGATGAGG‐3′ and 5′CCAGAGACGGAAATCCATCGCTCG‐3′.

### 2.10 RNA isolation and qRT‐PCR

RNA was isolated using RNeasy Protect Mini Kit (Qiagen #74124) with an on‐column DNase I digestion step with RNase‐Free DNase Kit (Qiagen #79254). It was reverse transcribed using iScript™ cDNA Synthesis Kit (Bio‐Rad #170‐8891). qRT‐PCR was performed with an ABI 7500 cycler (Applied Biosystems, Foster City, CA, USA). Relative expression levels of transcripts were calculated using the comparative CT method and normalized for GAPDH. The following primers were used: STAT1, ′5‐TGGTGAAATTGCAAGAGCTG‐3′ and ′5‐TGTGTGCGTACCCAAGATGT‐3′; IL‐6, ′5‐TGATGCACTTGCAGAAAACA‐3′ and ′5‐ACCAGAGGAAATTTTCAATAGGC‐3′; CXCL‐9, ′5‐CGATCCACTACAAATCCCTCA‐3′ and ′5‐TAGGCAGGTTTGATCTCCGT‐3′; CXCL‐10, ′5‐CTCATCCTGCTGGGTCTGAG‐3′ and ′5‐CCTATGGCCCTCATTCTCAC‐3′; CXCL‐11 ′5‐CTGCTGAGATGAACAGGAAGG‐3 ′and ′5‐CGCCCCTGTTTGAACATAAG‐3′.

### 2.11 Statistics

The normality of the data distribution was tested by Kolmogorov–Smirnov or D′Agostino–Pearson normality test. Significant differences in tumor load, multiplicity and size, colon length, colitis score, IHC stainings, qRT‐PCR data, and CIBERSORT score were calculated using unpaired *t*‐test (for normal data) and Mann–Whitney test (for non‐normal data). Multiple comparisons were calculated with one‐way ANOVA test and Tukey′s test, Bonferroni′s post‐test (for normal data) or Kruskal–Wallis test, and Dunn′s post‐test (for non‐normal data). Significant differences in weight loss were calculated by performing an area under the curve (AUC) calculation and testing the AUC by independent *t*‐test. Flow cytometry data from individual experiments were tested by a paired *t*‐test. Significant differences in tumor grade frequency, frequency of the four CMS subgroups within patient samples, and IL‐6 protein expression (assayed by IHC and quantitative histomorphometry with Definiens™ Tissue Studio software) were calculated by performing a χ^2^ test. Association between expression of STAT1 and CXCL‐9, CXCL‐10, and CXCL‐11 from human sample microarray data was calculated using Spearman′s rank coefficient correlation. The strength of correlation was determined as follows: *r* = 0.00–0.25—no correlation; *r* = 0.25–0.50—weak positive correlation; *r* = 0.50–0.75—positive correlation; *r* = 0.75–1.00—strong positive correlation. Survival analyses were carried out using log‐rank test. Significant differences between experimental groups were * = *P* *<* .05*,* ** = *P* *<* .01, and *** = *P* < .001.

### 2.12 Analysis of human samples

Recently published survival data, derived from STAT1 and STAT3 IHC stainings of human CRC tissue microarrays (Gordziel *et al*., 2013; Nivarthi *et al*., 2016), were used for sex stratification and evaluation of the prognostic value of tumor cell‐intrinsic nuclear STAT1 expression. Publicly available CRC microarray expression data (Guinney *et al*., 2015) were used to examine the expression of STAT1, CXCL‐9, CXCL‐10, and CXCL‐11 in patient samples. Stratification of the human sample microarray data into STAT1^high^ and STAT1^low^ groups was performed by fitting two Gaussian curves into the density distribution of STAT1 log_2_ expression using the R package mixtools. Samples were ranked according to their STAT1 log_2_ expression and ascribed posterior probabilities assigning them to either the STAT1^high^ or the STAT1^low^ group. The STAT1 log_2_ expression of the first sample with the probability of belonging to the STAT1^high^ group exceeding the probability of belonging to the STAT1^low^ group was chosen as threshold and set at 8.52. Overall, 1479 human CRC samples were stratified according to sex and STAT1 expression forming STAT1^high^ (534 samples) and STAT1^low^ (945 samples) groups. The CIBERSORT analysis was performed as described (Newman *et al*., 2015).

## 3 Results

### 3.1 Epithelial STAT1 is a sex‐specific promoter of acute colitis

We employed mice with specific deletion of STAT1 in intestinal epithelial cells (STAT1^∆IEC^) (el Marjou *et al*., 2004; Wallner *et al*., 2012) to investigate sex‐specific functions in colitis and colitis‐associated CRC. Deletion of STAT1 in intestinal epithelial cells (IECs) was confirmed by qRT‐PCR for STAT1 mRNA and by STAT1 IHC staining of formalin‐fixed and paraffin‐embedded whole gut preparations (Swiss rolls) (Crncec *et al*., 2015) of male (Fig. S1A,B) and female (Fig. S1C,D) STAT1^∆IEC^ mice. Lamina propria immune cells of STAT1^∆IEC^ mice readily displayed STAT1 expression, which demonstrated specific ablation in IECs (Fig. S1A,C). The mucosal architecture in small intestine and colon was not affected by STAT1 ablation. Enterocytes, goblet cells, enteroendocrine cells, Paneth cells, and Ki67^+^ cells in the intestinal crypts were present at normal numbers in STAT1^∆IEC^ mice (Fig. S2A–G).

**Figure 1:**
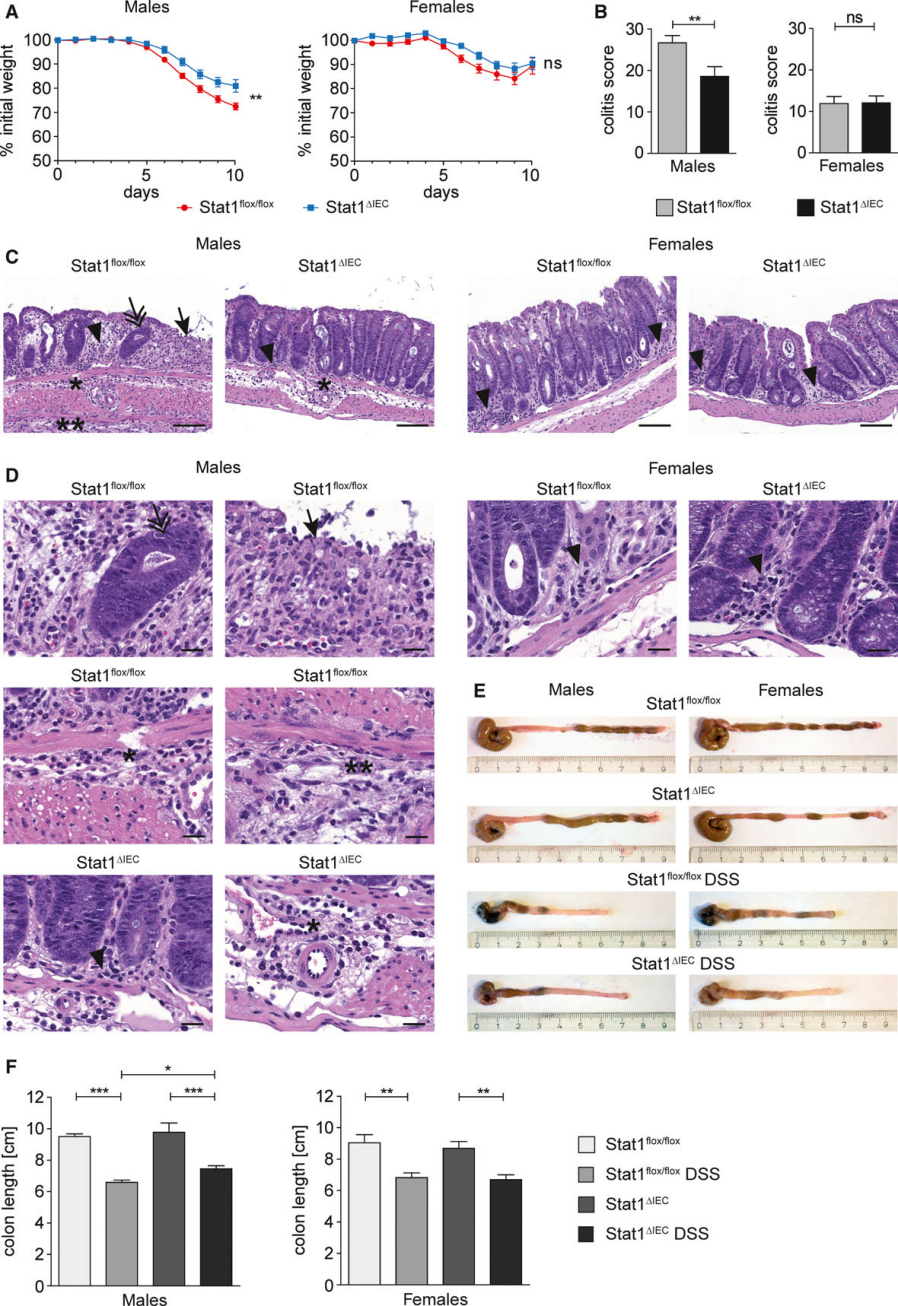
Epithelial STAT1 is a sex‐specific promoter of acute colitis. (A) Weight loss of DSS‐treated male (nine STAT1^flox/flox^, nine STAT1^∆IEC^) and female (eight STAT1^flox/flox^, nine STAT1^∆IEC^) mice. (B) Colitis score of DSS‐treated male (18 STAT1^flox/flox^, 18 STAT1^∆IEC^) and female (10 STAT1^flox/flox^, 15 STAT1^∆IEC^) mice. (C,D) H&E‐stained images for evaluation of colitis in DSS‐treated male and female STAT1^flox/flox^ and STAT1^∆IEC^ mice. Scale bar indicates 100 μm (C) or 20 μm (D). Arrow: complete erosion of epithelial surface; arrowhead: immune infiltration into the mucosa; double arrow: epithelial regenerative atypia simulating dysplasia; ӿ: immune infiltration into the submucosa; ӿӿ: immune infiltration into the subserosa. (E,F) Colon shortening in DSS‐treated male (nine STAT1^flox/flox^, 12 STAT1^∆IEC^) and female (eight STAT1^flox/flox^, seven STAT1^∆IEC^) mice (≥ 5 control mice per sex and genotype). Bars represent data ± SEM. ns: not significant.

We performed short‐term treatment of mice with DSS to investigate sex‐specific STAT1 functions in acute colitis. Male STAT1^∆IEC^ mice were partially protected from loss in body weight (Fig. 1A), and the colitis score was attenuated (Fig. 1B–D). Shortening of the colon length, which is indicative of the severity of colitis (Okayasu *et al*., 1990), was more pronounced in male STAT1^flox/flox^ mice than in male STAT1^∆IEC^ mice (Fig. 1E,F). In contrast, female STAT1^∆IEC^ mice were not protected from acute colitis (Fig. 1A–F). These data demonstrate that epithelial STAT1 is a male‐specific promoter of DSS‐induced colitis.

**Figure 2:**
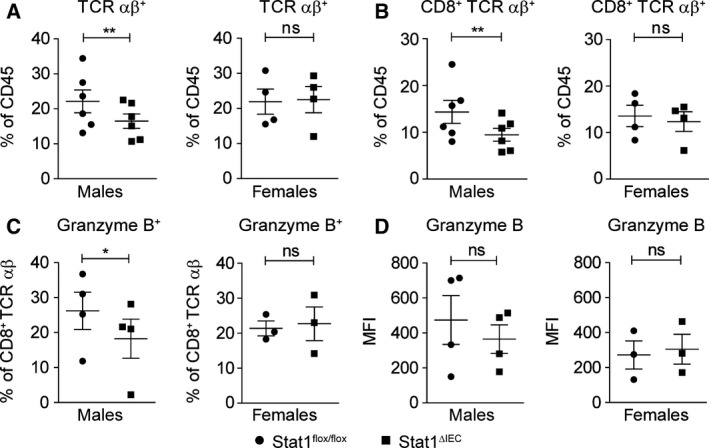
Epithelial STAT1 is a sex‐specific promoter of intraepithelial CD8^+^ T‐cell infiltration in acute colitis. (A–C) Flow cytometry data for intraepithelial infiltration of TCRαß^+^ T cells (A), CD8^+^ TCRαß^+^ T cells (B), and CD8^+^ TCRαß^+^ granzyme B^+^ T cells (C) into the mucosa of male and female STAT1^flox/flox^ and STAT1^∆IEC^ mice during DSS‐induced acute colitis. Each data point represents a biological replicate with cells pooled from three mice. (D) Mean fluorescence intensity for granzyme B expression in intraepithelial CD8^+^ T cells during DSS‐induced acute colitis. Each data point represents a biological replicate with cells pooled from three mice. ns: not significant.

Activated CD8^+^ T cells express cytotoxic molecules such as perforin and granzyme B that can damage the intestinal epithelium and aggravate colitis (Monteleone *et al*., 2012). Therefore, we characterized infiltration of lymphoid cells into the mucosa of DSS‐treated mice by flow cytometry analysis. These analyses revealed reduced intraepithelial TCRαβ^+^ and more specifically CD8^+^ TCRαβ^+^ T cells in male but not in female STAT1^∆IEC^ mice (Fig. 2A,B; Fig. S3A). The percentage of CD8^+^ TCRαβ^+^ T cells co‐expressing the activation marker granzyme B was also reduced in male STAT1^∆IEC^ mice (Fig. 2C; Fig. S3B) although intracellular granzyme B expression levels were not affected (Fig. 2D). Numbers of intraepithelial CD4^+^ TCRαβ^+^ T cells and CD8^+^ TCRγδ^+^ T cells were unchanged in male STAT1^∆IEC^ mice (Fig. S3C,D). T‐cell populations in the lamina propria were also unchanged in DSS‐treated male STAT1^∆IEC^ mice (Fig. S3E–I), but female STAT1^∆IEC^ mice showed increased numbers of CD4^+^ TCRαβ^+^ and CD8^+^ TCRαβ^+^ T cells (Fig. S3E–G). However, intraepithelial TCRαβ^+^ T‐cell populations were generally more abundant than lamina propria TCRαβ^+^ T cells (Fig. 2A; Fig. S3E). Moreover, the majority of CD8^+^ TCRαβ^+^ T cells were located in the intestinal epithelium in DSS‐treated mice (Fig. 2B; Fig. S3F). The data demonstrate that epithelial STAT1 is required for colitis‐associated intraepithelial infiltration of CD8^+^ TCRαβ^+^ granzyme B^+^ T cells in male but not in female mice.

### 3.2 Epithelial STAT1 is a sex‐specific inducer of T‐cell‐attracting chemokine expression in acute colitis

**Figure 3:**
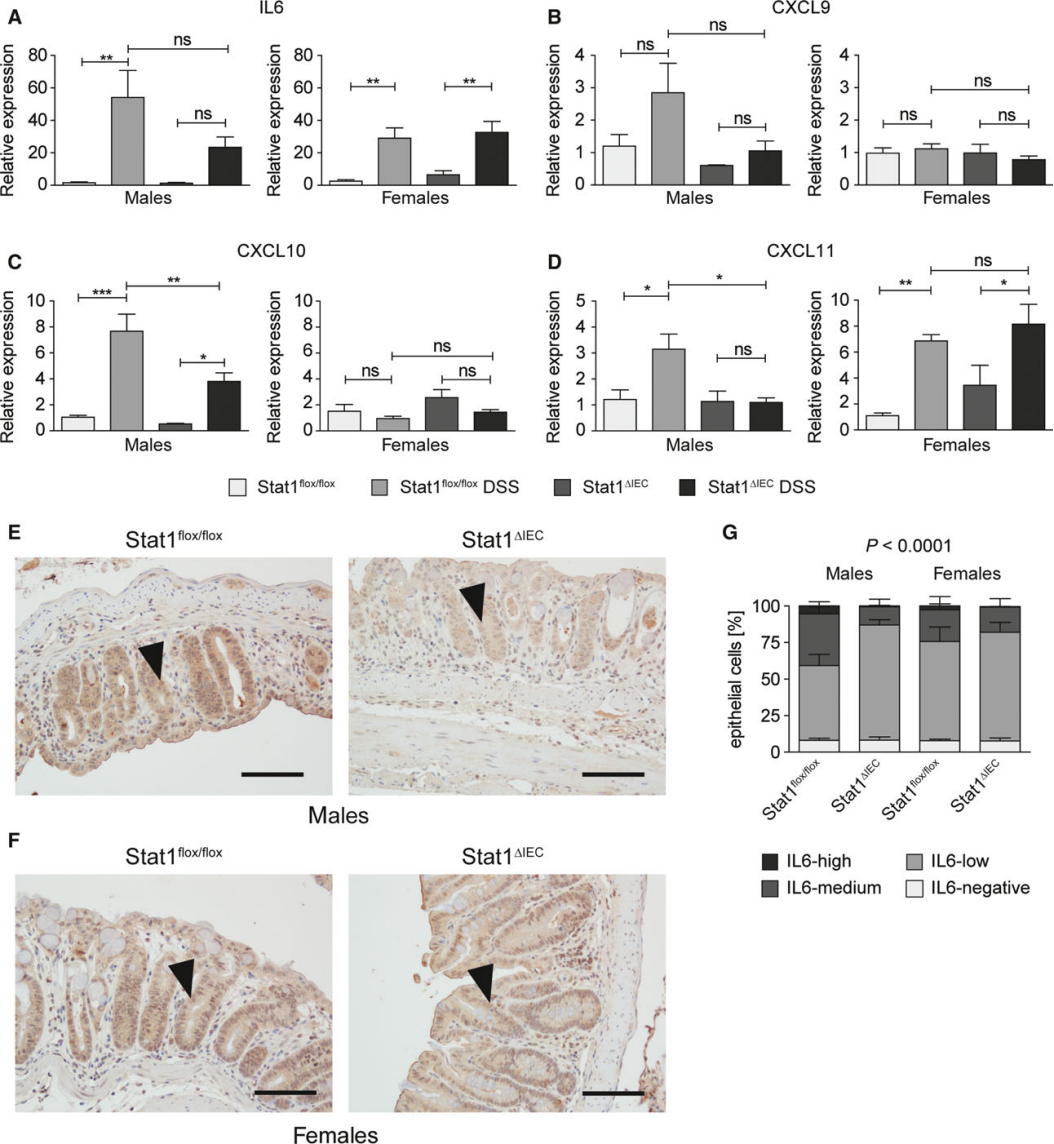
Epithelial STAT1 is a sex‐specific promoter of chemokine expression in acute colitis. (A–D) qRT‐PCR analysis for mRNA expression of IL‐6 (A), CXCL‐9 (B), CXCL‐10 (C), and CXCL‐11 (D) in isolated IECs of untreated versus DSS‐treated male and female STAT1^flox/flox^ and STAT1^∆IEC^ mice (4–6 biological replicates per treatment, sex, and genotype). Bars represent data ± SEM. ns: not significant. (E,F) Representative IHC stainings for IL‐6 (images; positive epithelial cells are indicated by arrowheads; scale bars indicate 50 μm) in DSS‐treated male (E) and female (F) STAT1^flox/flox^ and STAT1^∆IEC^ mice. (G) Quantification of positive epithelial cells with different staining intensities (bar diagrams; automated quantitative histomorphometry of four animals per genotype and sex) in DSS‐treated male and female STAT1^flox/flox^ and STAT1^∆IEC^ mice. Bars represent data ± SEM.

Our data indicate a sex‐specific requirement of epithelial STAT1 for chemoattraction of CD8^+^ TCRαβ^+^ T cells. IECs are a source for IL‐6, which is a T‐cell chemoattractant (Weissenbach *et al*., 2004), in DSS‐treated mice (Grivennikov *et al*., 2009), and IFN‐γ‐regulated chemokines CXCL‐9, CXCL‐10, and CXCL‐11 are secreted by epithelial cells and involved in inflammatory processes mediated by T cells (Marshall *et al*., 2017). Reduced induction of CXCL‐9 and CXCL‐10 was recently demonstrated in bulk colon tissue of DSS‐treated STAT1^∆IEC^ mice (Rauch *et al*., 2015) but this study did not discriminate between expression in epithelial or inflammatory cells and sex. We hypothesized that STAT1 would regulate the production of these T‐cell chemoattractants by IECs in a sex‐specific manner. Therefore, we purified IECs from DSS‐treated mice by MACS sorting and performed qRT‐PCR analysis. DSS treatment induced mRNA expression of IL‐6 in IECs of male and female STAT1^flox/flox^ mice, but the induction was slightly attenuated in male STAT1^∆IEC^ mice (Fig. 3A). Expression of CXCL‐9 mRNA was induced in male but not in female STAT1^flox/flox^ mice although differences did not reach significance. Similar to IL‐6, this induction was slightly attenuated in male STAT1^∆IEC^ mice (Fig. 3B). Expression of CXCL‐10 mRNA was strongly induced in male but not in female STAT1^flox/flox^ mice, albeit significantly attenuated in male STAT1^∆IEC^ mice (Fig. 3C). Expression of CXCL‐11 mRNA was induced by DSS in STAT1^flox/flox^ mice of both sexes but induction failed in IECs of male STAT1^∆IEC^ mice (Fig. 3D). These data revealed STAT1‐dependent sex‐specific effects of colitis on mRNA expression of T‐cell‐attracting chemokines CXCL‐9 and 10 in IECs. Moreover, STAT1 was found to be required for mRNA induction of CXCL‐11 in IECs of male but not of female mice. At the protein level, expression of IL‐6 was reduced in DSS‐treated male (Fig. 3E,G) but not female (Fig. 3F,G) STAT1^∆IEC^ mice. These data indicate a sex‐specific requirement of epithelial STAT1 for production of distinct T‐cell chemoattractants.

### 3.3 Epithelial STAT1 is a sex‐specific tumor suppressor in colitis‐associated CRC

**Figure 4:**
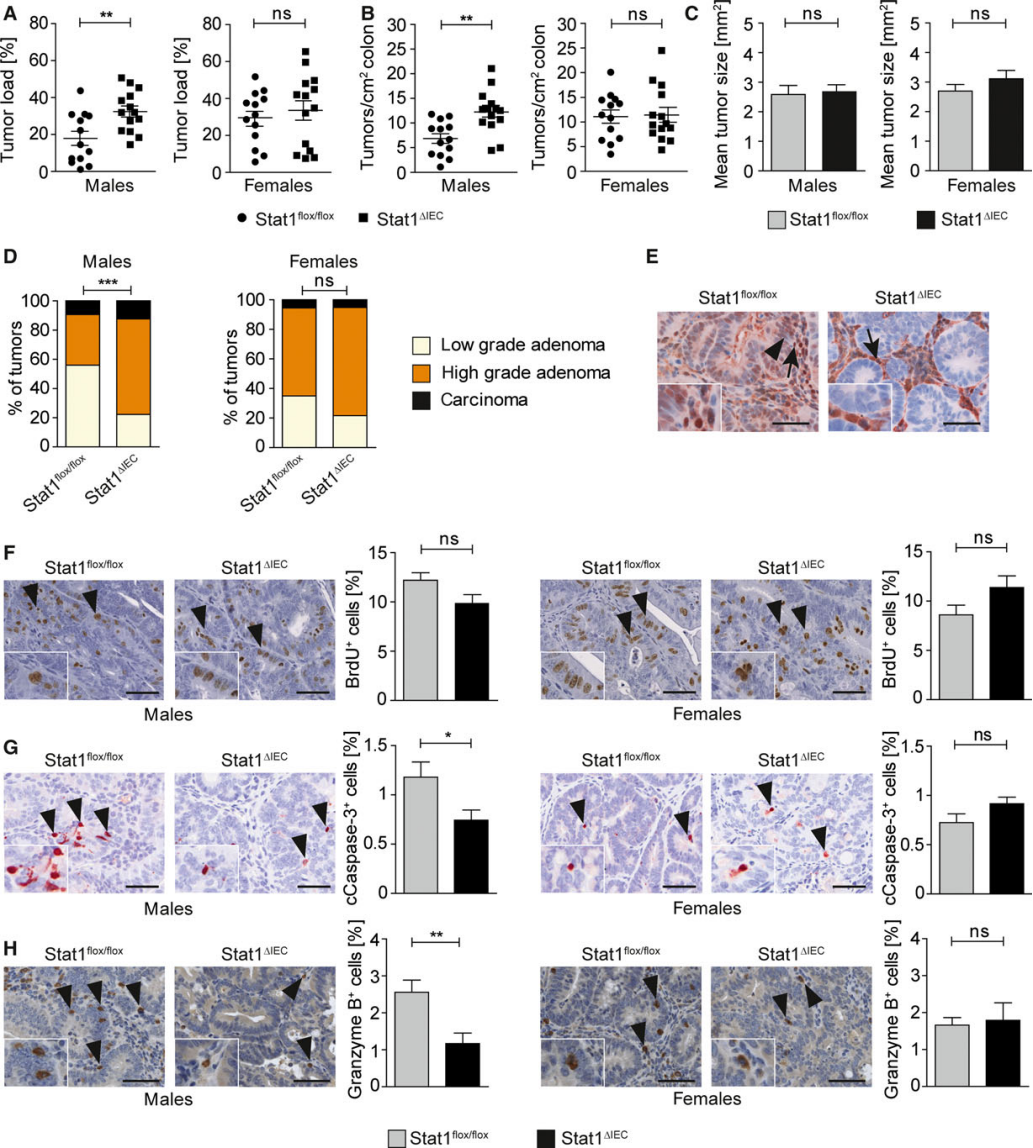
Epithelial STAT1 is a sex‐specific suppressor of colitis‐associated CRC. (A) Tumor load (% tumor area per total colon area) in male and female STAT1^flox/flox^ and STAT1^∆IEC^ mice. (B) Tumor multiplicity (number of tumors per cm^2^ colon) in male and female STAT1^flox/flox^ and STAT1^∆IEC^ mice. (C) Mean tumor size in male and female STAT1^flox/flox^ and STAT1^∆IEC^ mice (automated quantitative histomorphometry of ≥ 75 tumors per genotype in ≥ 13 animals per genotype). Bars represent data ± SEM. (D) Histopathological grading of colon tumors in male and female STAT1^flox/flox^ and STAT1^∆IEC^ mice (≥ 75 tumors per genotype in ≥ 13 animals per genotype). (E) IHC for STAT1 in tumors of STAT1^flox/flox^ and STAT1^∆IEC^ mice (insets show high magnifications; positive epithelial cells are indicated by arrowheads; positive stroma cells are indicated by arrows; scale bar indicates 50 μm). (F,G) BrdU IHC stainings for cell proliferation (F) and cleaved caspase‐3 IHC stainings for apoptosis (G) in colon tumors of male and female STAT1^flox/flox^ and STAT1^∆IEC^ mice (images; scale bar indicates 50 μm; positive cells are indicated by arrowheads and shown in detail by insets). Positive cells were quantified by automated quantitative histomorphometry (≥ 8 tumors per genotype in ≥ 3 animals per genotype). Bars represent data ± SEM. (H) IHC staining for granzyme B (images; scale bar indicates 100 μm; positive cells are indicated by arrowheads) and histomorphometric quantitation of granzyme B^+^ cells in colon tumors (≥ 9 tumors per genotype in ≥ 3 animals per genotype). Bars represent data ± SEM. ns: not significant.

DSS‐induced colitis is the tumor‐promoting condition in the azoxymethane/dextran sulfate sodium (AOM‐DSS) model of colitis‐associated CRC (Crncec *et al*., 2015), and we wondered if sex‐specific STAT1 effects in colitis and CD8^+^ T‐cell infiltration impact tumor formation. Therefore, colorectal tumors were induced in STAT1^∆IEC^ mice with AOM‐DSS (Crncec *et al*., 2015). Total tumor load was increased in male but not in female STAT1^∆IEC^ mice (Fig. 4A). Increased tumor load in STAT1^∆IEC^ males was due to increased tumor multiplicity (Fig. 4B), whereas the mean tumor size was not affected (Fig. 4C). Moreover, relative numbers of high‐grade adenomas were increased in male STAT1^∆IEC^ mice (Fig. 4D; Fig. S4A). IHC stainings confirmed loss of STAT1 and pY‐STAT1 (activated STAT1) in tumor cells of male and female STAT1^∆IEC^ mice (Fig. 4E, Fig. S4B,C), and no tumors that escaped STAT1 deletion were found. STAT1 activation was not prominent, and only a few tumor cells stained positive for pY‐STAT1 in STAT1^flox/flox^ mice (Fig. S4B,C). These data demonstrate a male‐specific tumor‐suppressive function of STAT1 in the formation and progression of colitis‐associated CRC in mice.

To get mechanistic insight, we analyzed sex‐specific STAT1 effects on tumor parameters, STAT3 activation, and expression of STAT1 target genes implicated in tumorigenesis. Numbers of proliferating cells were not significantly altered in both sexes (Fig. 4F), but apoptotic cells were reduced in tumors of male STAT1^∆IEC^ mice (Fig. 4G). Despite the described function of STAT1 in tumor angiogenesis (Meissl *et al*., 2015), no effect of STAT1 ablation on vessel density was observed (Fig. S5A). The closely related transcription factor STAT3 acts as an oncogene and conditional deletion in intestinal epithelial cells of mice interfered with AOM‐DSS‐induced CRC formation (Bollrath *et al*., 2009). Activation of STAT3 is frequently enhanced when STAT1 is deleted (Regis *et al*., 2008). Although numbers of STAT3‐expressing tumor cells were unchanged (Fig. S5B), we found increased STAT3 activation in STAT1^∆IEC^ tumors (Fig. S5C). This molecular effect was present in both sexes (Fig. S5C) suggesting that STAT3 does not contribute to STAT1‐dependent sex‐specific differences in tumorigenesis. Other STAT1 target genes such as p21 and c‐Myc were not differentially expressed in STAT1^flox/flox^ and STAT1^∆IEC^ tumors (Fig. S5D,E). Moreover, IHC staining revealed reduced numbers of granzyme B^+^ cells in tumors of male but not of female STAT1^∆IEC^ mice (Fig. 4H). This sex‐specific effect might blunt cytotoxic activity and lead to increased tumor load in male STAT1^∆IEC^ mice.

### 3.4 Tumor cell‐intrinsic nuclear STAT1 is a sex‐specific prognostic factor in human CRC

**Figure 5:**
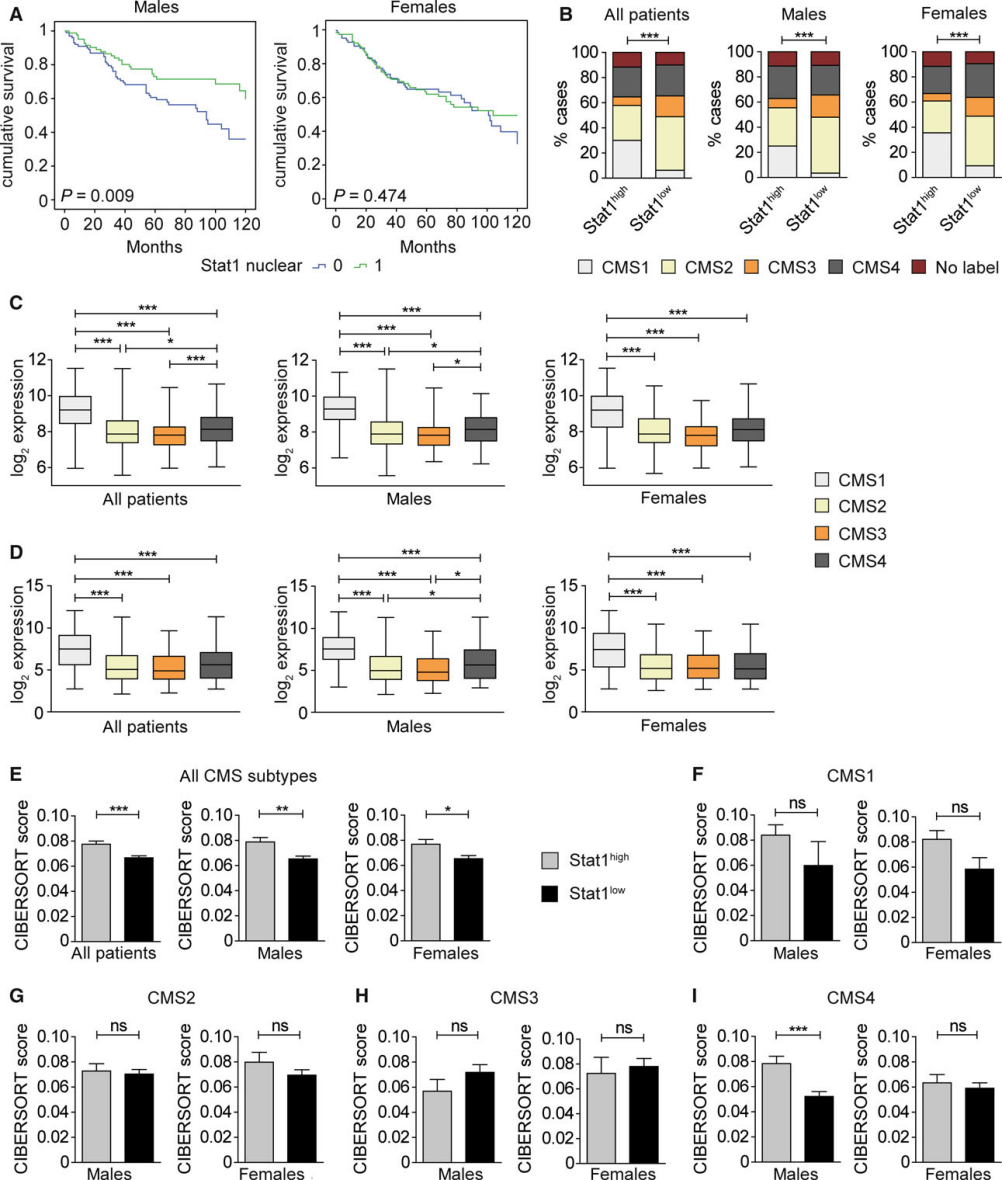
Tumor cell‐intrinsic nuclear STAT1 is a sex‐specific prognostic marker for human CRC. (A) Survival curves of male and female CRC patients with or without tumor cell‐intrinsic nuclear STAT1 expression. The analysis is based on published survival data (Gordziel *et al*., 2013) that were used for sex stratification. (B) STAT1 expression‐ and sex‐based stratification of CMS1‐4 subtypes of CRC (Guinney *et al*., 2015) . (C,D) STAT1 (C) and CXCL‐11 (D) log_2_ expression within CMS1‐4 subtypes of CRC (Guinney *et al*., 2015) in all patients and after sex stratification. (E) CIBERSORT analysis for CD8^+^ T‐cell infiltration in STAT1^high^ and STAT1^low^ CMS1—four subtypes of CRC without (all patients) and with sex stratification. (F‐I) CIBERSORT analysis for CD8^+^ T‐cell infiltration in sex‐stratified STAT1^high^ and STAT1^low^ CMS1 (F), CMS2 (G), CMS3 (H), and CMS4 (I) subtypes of CRC. Note that high STAT1 expression is indicative of CD8^+^ T‐cell infiltration in CMS4 CRC of male but not of female patients.

We recently performed STAT1 and STAT3 IHC stainings of human CRC tissue microarrays (Gordziel *et al*., 2013; Nivarthi *et al*., 2016). A distribution analysis of nuclear versus cytoplasmic STAT1 in cancer cells of these tissue microarrays revealed that most colorectal tumors were negative for STAT1 in both compartments or positive for STAT1 in the nucleus with or without cytoplasmic STAT1 expression (Fig. S6A). A similar distribution was obtained after sex stratification of CRC in men and women (Fig. S6B). Survival analyses demonstrated that tumor cell‐intrinsic nuclear STAT1 protein expression is a beneficial prognostic factor in CRC (Gordziel *et al*., 2013). However, sex stratification of the survival data revealed that this prognostic value is male‐specific (Fig. 5A). Tumor cell‐intrinsic cytoplasmic STAT1 was not identified as prognostic factor after sex stratification but there was a trend toward favorable prognosis in male patients (Fig. S6C). We also demonstrated that cytoplasmic but not nuclear STAT3 represents a prognostic factor in human CRC (Gordziel *et al*., 2013). Stratification of these data revealed no sex bias for the prognostic value of cytoplasmic STAT3 (Fig. S6D), although there was a trend toward favorable prognosis in male patients. Moreover, neither nuclear STAT3 nor concomitant nuclear STAT1 and nuclear STAT3 represented a sex‐specific prognostic factor (Fig. S6E,F). A sex‐specific correlation with clinicopathological parameters showed that tumor grading or tumor staging was not prognostic in male and female CRC patients (Fig. S7A,B). However, vein invasion of tumor cells and lymph node metastasis was a significant factor for bad prognosis in both sexes (Fig. S7C,D). Metastasis is the cause for mortality in about 90% of cancer patients (Spano *et al*., 2012). Therefore, we investigated if the good prognosis of male patients with prominent STAT1 expression in cancer cells is due to reduced vein invasion or lymph node metastasis. Interestingly, we could neither identify a significant correlation between tumor cell‐intrinsic nuclear STAT1 expression and vein invasion (Table S1) nor lymph node metastasis (Table S2) in both sexes. This suggests that tumor cell‐intrinsic STAT1 is implicated in the invasion–metastasis cascade of male CRC patients at a stage beyond vein invasion. In summary, our data demonstrate that expression of tumor cell‐intrinsic nuclear STAT1 protein is a male‐specific prognostic factor in human CRC. Moreover, sex stratification can significantly refine the prognostic value of STAT1 in CRC patients.

### 3.5 Sex‐specific correlation between STAT1 expression and abundance of CD8^+^ T cells in the CMS4 subgroup of human CRC

We next stratified publicly available CRC gene expression data for the recently described consensus molecular subtypes (CMS) of CRC (Guinney *et al*., 2015) into STAT1^high^‐ and STAT1^low^‐expressing subgroups (Fig. S8) to assess correlations between STAT1, IL‐6, CXCL‐9, CXCL‐10, CXCL‐11 cytokine/chemokine expression, CD8^+^ T‐cell infiltration, and sex. The percentage of CMS1 tumors was higher within the STAT1^high^ subgroup compared to the STAT1^low^ subgroup irrespective of sex, which is most likely due to the strong immune cell infiltration of this subtype (Guinney *et al*., 2015). In contrast, the percentages of CMS2 and CMS3 were lower in the STAT1^high^ subgroup and the CMS4 subtype was equally distributed between STAT1^high^ and STAT1^low^ subgroups (Fig. 5B). Expression of STAT1 did not correlate with IL‐6 (Fig. S9B–D) but correlated positively with T‐cell‐attracting chemokines CXCL‐9, CXCL‐10, and CXCL‐11 in all four CMS subtypes irrespective of the sex (Fig. S10 ,S11,S12B–D,B–D,A–C). STAT1, IL‐6, CXCL‐9, CXCL‐10, and CXCL‐11 were most prominently expressed in CMS1 and CMS4 subtypes (Fig. 5C–D; Fig. S9, S10, S11A). The CMS4 subtype, which is characterized by invasiveness and bad prognosis (Guinney *et al*., 2015), showed a sex‐specific increase in STAT1 and CXCL‐11 expressions. Both genes were expressed at significantly higher levels in CMS4 than in CMS2 or CMS3 subtypes of male but not of female patients (Fig. 5C,D). In contrast, IL‐6, CXCL‐9, and CXCL‐10 were significantly increased in CMS4 tumors of both sexes (Fig. S9, S10, S11A). Importantly, immune phenotyping by CIBERSORT (Newman *et al*., 2015) demonstrated that STAT1 expression correlated with CD8^+^ T‐cell infiltration in CRC (Fig. 5E). Stratification of these data according to the CMS subtype (Fig. 5F–I) revealed a male‐specific positive correlation between STAT1 expression and CD8^+^ T‐cell infiltration in CMS4 (Fig. 5I). These data suggest that STAT1 regulates CD8^+^ T‐cell infiltration in CMS4 tumors of male but not of female patients.

## 4 Discussion

The identity of sex‐specific molecular factors in cancer formation and progression remains scarce (Clocchiatti *et al*., 2016). We investigated sex‐specific STAT1 functions in colitis and colitis‐associated CRC using mice with specific STAT1 deletion in intestinal epithelial cells (STAT1^∆IEC^). Male but not female STAT1^∆IEC^ mice were more resistant to DSS‐induced colitis than sex‐matched controls and displayed reduced intraepithelial infiltration of CD8^+^ TCRαβ^+^ granzyme B^+^ T cells. The AOM‐DSS protocol for induction of colitis‐associated CRC resulted in increased intestinal tumor load in male but not in female STAT1^∆IEC^ mice. A sex‐specific stratification of human CRC patients corroborated the data obtained in mice and revealed that reduced tumor cell‐intrinsic nuclear STAT1 protein expression is a poor prognostic factor in men but not in women. Our data demonstrate that epithelial STAT1 is a sex‐specific tumor suppressor in CRC of mice and humans. To our knowledge, epithelial STAT1 is the first factor that acts as a male‐specific tumor suppressor in CRC.

DSS‐induced colitis was attenuated in male STAT1^∆IEC^ mice. This observation contrasts a recent study that showed aggravated colitis in DSS‐treated STAT1^∆IEC^ mice (Chiriac *et al*., 2017). We suspect that different microbiota conditions in the animal facilities account for this discrepancy. Concomitant with attenuated colitis, intraepithelial infiltration of CD8^+^ TCRαβ^+^ granzyme B^+^ T cells was reduced in male STAT1^∆IEC^ mice. CXCL‐9, CXCL‐10, and CXCL‐11 chemokines attract CXCR‐3‐expressing CD8^+^ T cells and promote Th1 responses during inflammatory conditions (Groom and Luster, 2011). These chemokines are regulated by IFN‐γ and produced by epithelial cells (Marshall *et al*., 2017). We observed a DSS‐mediated induction of IL‐6, CXCL‐9, CXCL‐10, and CXCL‐11 mRNA expression levels in IECs of male control mice. This induction was blunted in male STAT1^∆IEC^ mice, which might account for the reduced infiltration of CD8^+^ TCRαβ^+^ granzyme B^+^ T cells. We confirmed blunted IL‐6 induction in male STAT1^∆IEC^ mice at the protein level because mRNA data were ambiguous and did not reach significance. Only IL‐6 and CXCL‐11 mRNA were induced by DSS in epithelial cells of female control mice, and induction was maintained in sex‐matched STAT1^∆IEC^ mice, which was confirmed for IL‐6 at the protein level. These data suggest a male‐specific requirement for epithelial STAT1 in IL‐6, CXCL‐9, CXCL‐10, and CXCL‐11 expression during DSS‐induced colitis. The CXCL‐11 gene contains a frameshift mutation in the C57BL/6 mouse strain (Sierro *et al*., 2007), but the promoter is still regulated by IFN‐γ (Benson and Ernst, 2009). Therefore, blunted CXCL‐11 expression cannot explain reduced infiltration of CD8^+^ TCRαβ^+^ granzyme B^+^ T cells in STAT1^∆IEC^ mice (which are C57BL/6). However, CXCL‐10 has overlapping functions with CXCL‐11 (Groom and Luster, 2011) and impaired induction might contribute to reduced infiltration.

Colitis is the tumor‐promoting condition in the AOM‐DSS model (Crncec *et al*., 2015). It is therefore counterintuitive that the tumor load was increased in AOM‐DSS‐treated male STAT1^∆IEC^ mice although severity of DSS‐induced colitis was reduced. However, inflammation has a dual role in tumorigenesis and it depends on the immune cell composition whether tumor formation is promoted or inhibited (Monteleone *et al*., 2012). CD8^+^ T cells can aggravate tissue damage, which may sustain a tumor‐promoting chronic inflammation (Monteleone *et al*., 2012), but they also release different cytotoxic molecules such as IFN‐γ which kill dysplastic cells (Restifo *et al*., 2012). Accordingly, antibody‐mediated depletion of CD8^+^ T cells led to increased tumor load in AOM‐DSS‐treated mice (Pastille *et al*., 2014). In addition, AOM‐DSS‐treated mice with ectopic Smad7 expression in T cells showed more severe colitis but reduced tumor load, which was associated with increased tumor immune surveillance by CD8^+^ T cells (Rizzo *et al*., 2011). This suggests that tumor suppression by CD8^+^ T cells prevails over tumor‐promoting effects of colitis in the AOM‐DSS model. Therefore, reduced numbers of intraepithelial CD8^+^ TCRαβ^+^ granzyme B^+^ T cells during DSS‐induced colitis might have created an immune‐privileged microenvironment in male STAT1^∆IEC^ mice that led to a higher tumor load despite of reduced colitis.

Reduced intraepithelial granzyme B^+^‐cell infiltration during DSS‐induced colitis was preserved in AOM‐DSS‐induced tumors of male STAT1^∆IEC^ mice, which contained lower numbers of granzyme B^+^ cells than sex‐matched control tumors. This occurred without implication of intratumoral chemokine expression because RNA‐seq analysis did not reveal reduced mRNA levels for CXCL‐9, CXCL‐10, and CXCL‐11 in bulk tumor tissue of male STAT1^∆IEC^ mice (data not shown). Low numbers of intratumoral granzyme‐expressing CD8^+^ T cells are associated with bad prognosis of human CRC (Angell and Galon, 2013; Galon *et al*., 2006, 2013). Consistently, reduced infiltration of intratumoral granzyme‐expressing cells correlated with an increased tumor load and a higher percentage of high‐grade tumors in male STAT1^∆IEC^ mice. Moreover, apoptosis was reduced which might be due to impaired granzyme B^+^‐cell‐mediated killing.

We recently employed IHC staining of tissue microarrays and identified nuclear STAT1 expression in cancer cells as a beneficial prognostic factor for CRC patients (Gordziel *et al*., 2013). Prompted by our sex‐specific observations in mice, we stratified these data according to sex. This strategy significantly improved the prognostic value of STAT1 in the male cohort but abolished it in the female cohort indicating that tumor cell‐intrinsic nuclear STAT1 suppresses CRC progression in male but not in female patients. Moreover, we identified a male‐specific positive correlation between STAT1 expression and CD8^+^ T‐cell infiltration in the CMS4 subtype of human CRC (Guinney *et al*., 2015) using CIBERSORT analysis of bulk gene expression data (Newman *et al*., 2015). It has to be shown if tumor cell‐intrinsic STAT1 is responsible for this sex‐specific effect because bulk gene expression data do not discriminate between tumor and stromal cells. CMS4 tumors displayed also sex‐specific differences in STAT1/CXCL‐11 expression, but these differences were likely too subtle to explain enhanced male‐specific CD8^+^ T‐cell infiltration.

In summary, we identified epithelial STAT1 as a molecular factor that affects CRC formation in a sex‐specific manner. Consequently, the prognostic value of tumor cell‐intrinsic STAT1 improved significantly after sex stratification of CRC patients. Our data also recommend sex stratification prior to evaluation of therapeutic efficacy of JAK‐STAT inhibitors in IBD patients (Sandborn *et al*., 2012, 2014) to account for sex‐specific STAT1 effects on inflammation.

## Author contributions

IC, RE, and KF involved in conceptualization; IC and MMo involved in methodology; IC, MMo, CG, JS, IS, SM, PP, and GT investigated the study; RE wrote the original draft; IC, MMo, JS, LK, MM, BS, and RE wrote, reviewed and edited the manuscript; MM and RE acquired the funding; MS, LK, EC, MM, BS, EB, TM, and JS provided the resources; RE supervised the study.

## Supporting information


**Fig. S1.** Specific deletion of STAT1 in intestinal epithelial cells of STAT1∆IEC mice.
**Fig. S2.** STAT1 is not required for development of the intestinal architecture.
**Fig. S3.** Gating strategy for flow cytometry analyses and lamina propria immune cells in STAT1flox/flox and STAT1∆IEC mice during DSS‐induced colitis.
**Fig. S4.** Activation status of STAT1 in AOM‐DSS‐induced tumors.
**Fig. S5.** Blood vessel formation and STAT1 target gene expression in AOM‐DSS‐induced colorectal tumors.
**Fig. S6.** Tumor cell‐intrinsic cytoplasmic STAT1 or nuclear STAT3 is not a sex‐specific prognostic marker for human CRC.
**Fig. S7.** Vein invasion and lymph node metastases are negative prognostic markers for human CRC in male and female patients.
**Fig. S8.** Strategy for stratification of human CMS1‐4 subtypes of CRC into STAT1high‐ and STAT1low‐expressing subgroups.
**Fig. S9.** IL‐6 expression does not correlate with STAT1 expression in human CRC.
**Fig. S10.** CXCL‐9 expression correlates positively with STAT1 expression in human CRC.
**Fig. S11.** CXCL‐10 expression correlates positively with STAT1 expression in human CRC.
**Fig. S12.** CXCL‐11 expression correlates positively with STAT1 expression in human CRC.
**Table S1.** No correlation of tumor cell‐intrinsic nuclear STAT1 expression and vein invasion.
**Table S2.** No correlation of tumor cell‐intrinsic nuclear STAT1 expression and lymph node metastasis.
