# Supplementary material for: STAT1 is a sex‐specific tumor suppressor in colitis‐associated colorectal cancer
Source: Mol Oncol. 2018 Feb 20;12(4):514–28. doi: 10.1002/1878-0261.12178 (PMC5891040; doi:10.1002/1878-0261.12178)
Supplement: Supplementary file 1 — Fig. S1. Specific deletion of STAT1 in intestinal epithelial cells of STAT1∆IEC mice. Fig. S2. STAT1 is not required for development of the intestinal architecture. Fig. S3. Gating strategy for flow cytometry analyses and lamina propria immune cells in STAT1flox/flox and STAT1∆IEC mice during DSS‐induced colitis. Fig. S4. Activation status of STAT1 in AOM‐DSS‐induced tumors. Fig. S5. Blood vessel formation and STAT1 target gene expression in AOM‐DSS‐induced colorectal tumors. Fig. S6. Tumor cell‐intrinsic cytoplasmic STAT1 or nuclear STAT3 is not a sex‐specific prognostic marker for human CRC. Fig. S7. Vein invasion and lymph node metastases are negative prognostic markers for human CRC in male and female patients. Fig. S8. Strategy for stratification of human CMS1‐4 subtypes of CRC into STAT1high‐ and STAT1low‐expressing subgroups. Fig. S9. IL‐6 expression does not correlate with STAT1 expression in human CRC. Fig. S10. CXCL‐9 expression correlates positively with STAT1 expression in human CRC. Fig. S11. CXCL‐10 expression correlates positively with STAT1 expression in human CRC. Fig. S12. CXCL‐11 expression correlates positively with STAT1 expression in human CRC. Table S1. No correlation of tumor cell‐intrinsic nuclear STAT1 expression and vein invasion. Table S2. No correlation of tumor cell‐intrinsic nuclear STAT1 expression and lymph node metastasis. [file MOL2-12-514-s001.pdf]

Supplemental Figure legends

Fig. S1. Specific deletion of STAT1 in intestinal epithelial cells of STAT1$^{\Delta IEC}$ mice. (A-D) Deletion of STAT1 in intestinal epithelial cells of male (A, B) and female (C, D) STAT1$^{\Delta IEC}$ mice was confirmed by IHC for total STAT1 (A, C; ≥ 2 mice per genotype; upper images represent small intestine; lower images represent colon; insets show high magnifications; positive epithelial cells are indicated by arrowheads; positive lamina propria cells are indicated by arrows; scale bar indicates 100 µm) and qRT-PCR for STAT1 from isolated epithelial cells (B, D; bars represent data +/- SEM ; ≥ 5 mice per genotype).

Fig. S2. STAT1 is not required for development of the intestinal architecture. (A-G) Alcian blue staining for Goblet cells (A, E), IHC staining for enteroendocrine cells (synaptophysin) (B, F), Paneth cells (lysozyme) (C) and cell proliferation (Ki67) (D, G) in male and female STAT1$^{flox/flox}$ and STAT1$^{\Delta IEC}$ mice (images in A-D represent small intestine; images in E-G represent colon; insets show high magnifications; positive cells are indicated by arrowheads; scale bars indicate 100 µm). Positive cells were quantified manually (bar diagrams; ≥ 9 villi/crypts in ≥ 2 animals per genotype). Bars represent data +/- SEM. ns: not significant.

Fig. S3. Gating strategy for flow cytometry analyses and lamina propria immune cells in STAT1$^{flox/flox}$ and STAT1$^{\Delta IEC}$ mice during DSS-induced colitis. (A, B) Representative gating strategy for intraepithelial and lamina propria CD8$^+$ TCRαß$^+$ and CD4$^+$ TCRαß$^+$ T cells (A) and CD8$^+$ TCRαß$^+$ Granzyme B$^+$ T cells (B) of STAT1$^{flox/flox}$ and STAT1$^{\Delta IEC}$ mice during DSS-induced colitis. (C, D) Flow cytometry quantification of intraepithelial CD4$^+$ TCRαß$^+$ (C) and CD8$^+$ TCRγδ$^+$ T cells (D) of male and female STAT1$^{flox/flox}$ and STAT1$^{\Delta IEC}$ mice during DSS-induced colitis. Each data point represents a biological replicate with cells pooled from 3 mice. (E-H) Flow cytometry quantification of TCRαß$^+$ (E), CD8$^+$ TCRαß$^+$ (F), CD4$^+$ TCRαß$^+$ T cells (G) and CD8$^+$ TCRαß$^+$ Granzyme B$^+$ T cells (H) in the lamina propria of male and female STAT1$^{flox/flox}$ and STAT1$^{\Delta IEC}$ mice during DSS-induced colitis. Each data point represents a biological replicate with cells pooled from 3 mice. (I) Mean fluorescence intensity for expression of Granzyme B within the CD8$^+$ TCRαß$^+$ T cell population in the lamina propria of male and female STAT1$^{flox/flox}$ and STAT1$^{\Delta IEC}$ mice during DSS-induced colitis. Each data point represents a biological replicate with cells pooled from 3 mice. ns: not significant.

Fig. S4. Activation status of STAT1 in AOM-DSS-induced tumors. (A) Representative images for low grade adenomas, high grade adenomas and carcinomas in STAT1$^{flox/flox}$ and STAT1$^{\Delta IEC}$ mice. Tumors in Swiss rolls were stained with H&E and graded according to several criteria. Low grade adenomas: loss or significant decrease of goblet cells, cytoplasmic hyperchromasia

of nuclei which show regular basal orientation and are not markedly enlarged (arrows). High grade adenomas: back to back glands without intervening stroma, total loss of polarity by atypical cells (arrows), sometimes mucin-producing cells that are forming lumens, technically appearing cribriform. Carcinomas: nuclei round and vesicular, tumor glands do not show appreciable numbers of goblet cells, glands are often fused with secondary lumina and show an increasing complexity, invasion through the muscularis mucosae (arrow) into the submucosa so that the invasion front of the tumor can be seen in the submucosa and frequently in close proximity to submucosal blood vessels, frequent presence of desmoplasia. Insets show high magnifications. Scale bars indicate 200 μm. (B, C) IHC stainings for activated pY-STAT1 (images; insets show high magnifications; positive epithelial cells are indicated by arrowheads; positive stroma cells are indicated by arrows; scale bars indicate 50 μm) and quantification of positive cells (bar diagrams; automated quantitative histomorphometry of ≥ 9 tumors per genotype in ≥ 3 animals per genotype) in AOM-DSS-induced colorectal tumors of male (B) and female (C) $STAT1^{flox/flox}$ and $STAT1^{\Delta IEC}$ mice. Bars represent data +/- SEM. nd: not detectable. ns: not significant.

Fig. S5. Blood vessel formation and STAT1 target gene expression in AOM-DSS-induced colorectal tumors. (A) IHC stainings for vessels (endomucin) in AOM-DSS-induced colorectal tumors of male and female $STAT1^{flox/flox}$ and $STAT1^{\Delta IEC}$ mice tumors (images; insets show high magnifications; vessels are indicated by arrowheads; scale bar indicates 50 μm) and quantification of tumor vessels (bar diagrams; automated quantitative histomorphometry of ≥ 9 tumors per genotype in ≥ 3 animals per genotype). (B-E) IHC stainings for total STAT3 (B), activated pY-STAT3 (C), p21 (D) and c-Myc (E) in tumors (images; insets show high magnifications; positive nuclei are indicated by arrowheads; scale bar indicates 50 μm) and quantification of positive cells (automated quantitative histomorphometry of ≥ 9 tumors per genotype in ≥ 3 animals per genotype) in AOM-DSS-induced colorectal tumors of male and female $STAT1^{flox/flox}$ and $STAT1^{\Delta IEC}$ mice. Bars represent data +/- SEM. ns: not significant.

Fig. S6. Tumor cell-intrinsic cytoplasmic STAT1 or nuclear STAT3 is not a sex-specific prognostic maker for human CRC. (A, B) Distribution of tumor cell-intrinsic STAT1 in cytoplasmic and nuclear cellular compartments of human CRC without (A) and with sex stratification (B). The data were obtained from published IHC-stained tissue microarrays of human CRC samples (Gordziel et al., 2013). (C-F) Survival curves of male and female CRC patients stratified for tumor cell-intrinsic cytoplasmic STAT1 protein (C), tumor cell-intrinsic cytoplasmic STAT3 protein (D), tumor cell-intrinsic nuclear STAT3 protein (E) or concomitant tumor cell-intrinsic nuclear STAT1 and STAT3 proteins (F). All data were derived from

published survival data (Gordziel et al., 2013) after sex stratification. The log rank p values are indicated in the lower left-hand corner of the graphs.

Fig. S7. Vein invasion and lymph node metastases are negative prognostic markers for human CRC in male and female patients. (A-D) Survival curves of male and female CRC patients stratified for tumor grade (A), tumor stage (B), vein invasion (C) and lymph node metastases (D). All data were derived from published survival data (Gordziel et al., 2013) after sex stratification. The log rank p values are indicated in the lower left-hand corner of the graphs.

Fig. S8. Strategy for stratification of human CMS1-4 subtypes of CRC into $STAT1^{high}$- and $STAT1^{low}$-expressing subgroups. Publicly available CRC gene expression data for the recently described CMS subtypes of CRC (Guinney et al., 2015) were used to stratify tumors according to STAT1 expression. The threshold between the $STAT1^{high}$ and $STAT1^{low}$ subgroups was set at 8.52. For details see materials and methods.

Fig. S9. IL-6 expression does not correlate with STAT1 expression in human CRC. (A) IL-6 log_2 expression within CMS1-4 subtypes of CRC (Guinney et al., 2015) in all patients and after sex stratification. (B-D) Correlation of STAT1 and IL-6 expression in CMS1-4 subtypes in all patients (B) and after sex stratification in male (C) and female (D) patients. The Spearman´s rank correlation coefficient (r) is indicated in the upper left-hand corner of each graph.

Fig. S10. CXCL-9 expression correlates positively with STAT1 expression in human CRC. (A) CXCL-9 log_2 expression within CMS1-4 subtypes of CRC (Guinney et al., 2015) in all patients and after sex stratification. (B-D) Correlation of STAT1 and CXCL-9 expression in CMS1-4 subtypes in all patients (B) and after sex stratification in male (C) and female (D) patients. The Spearman´s rank correlation coefficient (r) is indicated in the upper left-hand corner of each graph.

Fig. S11. CXCL-10 expression correlates positively with STAT1 expression in human CRC. (A) CXCL-10 log_2 expression within CMS1-4 subtypes of CRC (Guinney et al., 2015) in all patients and after sex stratification. (B-D) Correlation of STAT1 and CXCL-10 expression in CMS1-4 subtypes in all patients (B) and after sex stratification in male (C) and female (D) patients. The Spearman´s rank correlation coefficient (r) is indicated in the upper left-hand corner of each graph.

Fig. S12. CXCL-11 expression correlates positively with STAT1 expression in human CRC. (A-C) Correlation of STAT1 and CXCL-11 expression in CMS1-4 subtypes of CRC (Guinney

et al., 2015) in all patients (A) and after sex stratification in male (B) and female (C) patients. The Spearman´s rank correlation coefficient (r) is indicated in the upper left-hand

References

Gordziel, C., Bratsch, J., Moriggl, R., Knosel, T., Friedrich, K., 2013. Both STAT1 and STAT3 are favourable prognostic determinants in colorectal carcinoma. Br J Cancer 109, 138-146.

Guinney, J., Dienstmann, R., Wang, X., de Reynies, A., Schlicker, A., Soneson, C., Marisa, L., Roepman, P., Nyamundanda, G., Angelino, P., Bot, B.M., Morris, J.S., Simon, I.M., Gerster, S., Fessler, E., De Sousa, E.M.F., Missiaglia, E., Ramay, H., Barras, D., Homicsko, K., Maru, D., Manyam, G.C., Broom, B., Boige, V., Perez-Villamil, B., Laderas, T., Salazar, R., Gray, J.W., Hanahan, D., Tabernero, J., Bernards, R., Friend, S.H., Laurent-Puig, P., Medema, J.P., Sadanandam, A., Wessels, L., Delorenzi, M., Kopetz, S., Vermeulen, L., Tejpar, S., 2015. The consensus molecular subtypes of colorectal cancer. Nat Med 21, 1350-1356.

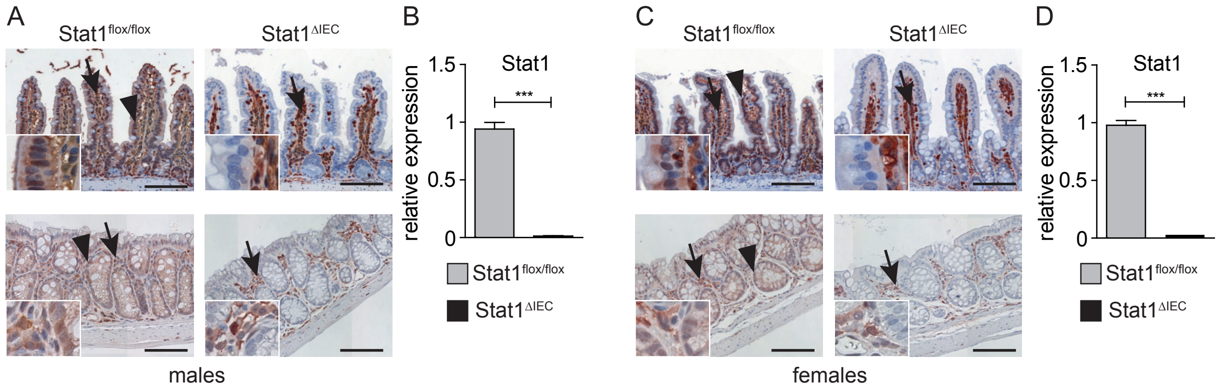

Supplemental Figure 1

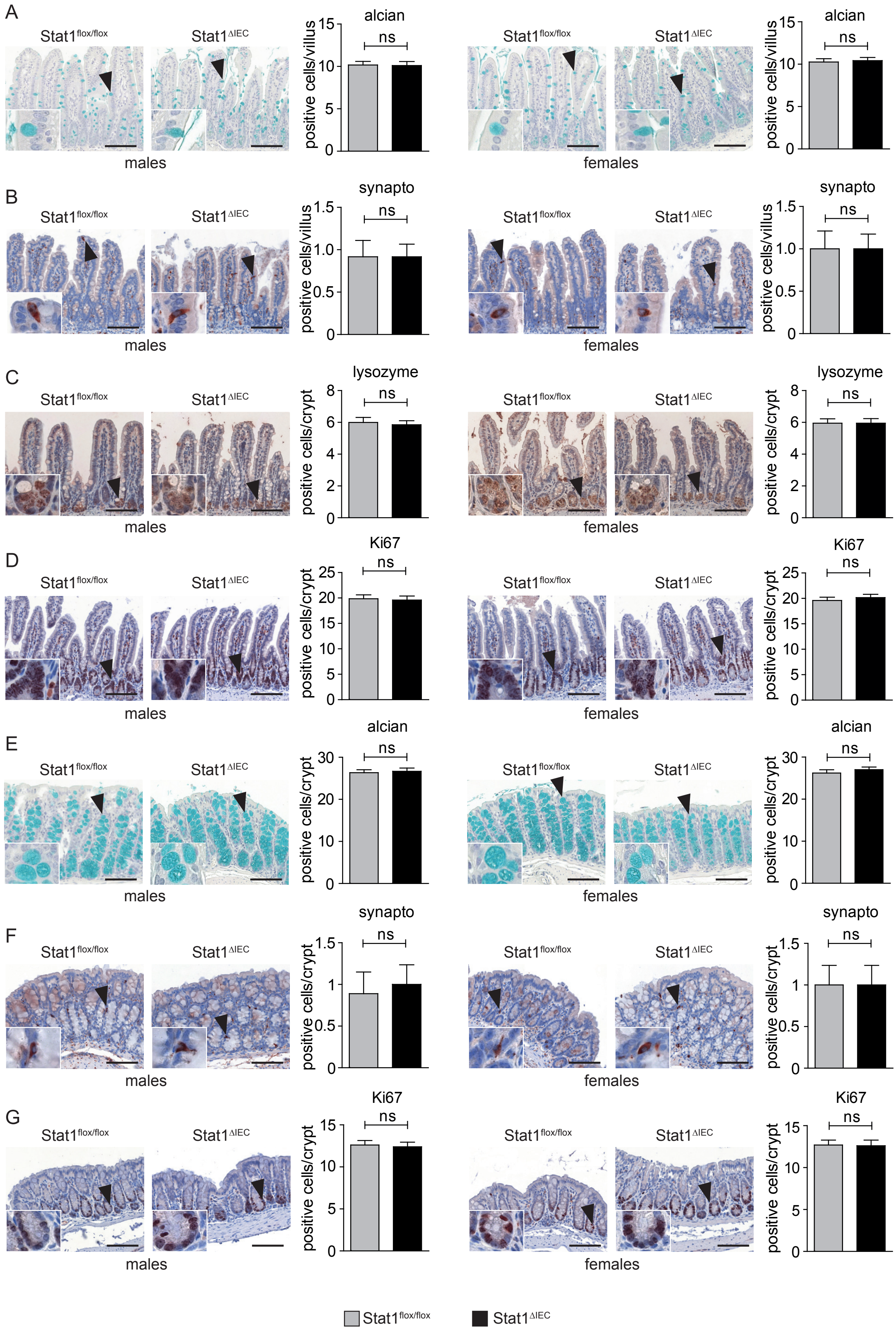

Supplemental Figure 2

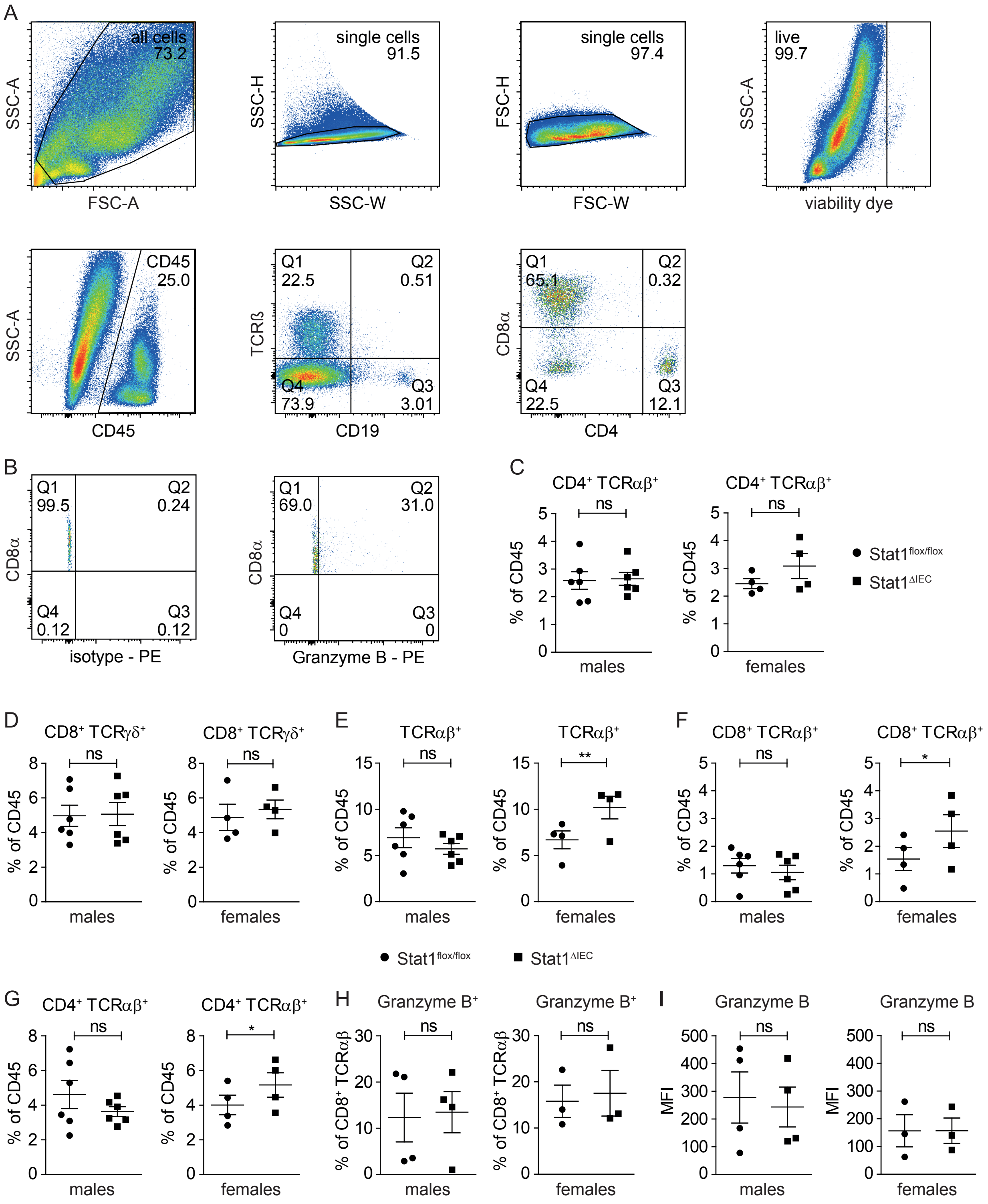

Supplemental Figure 3

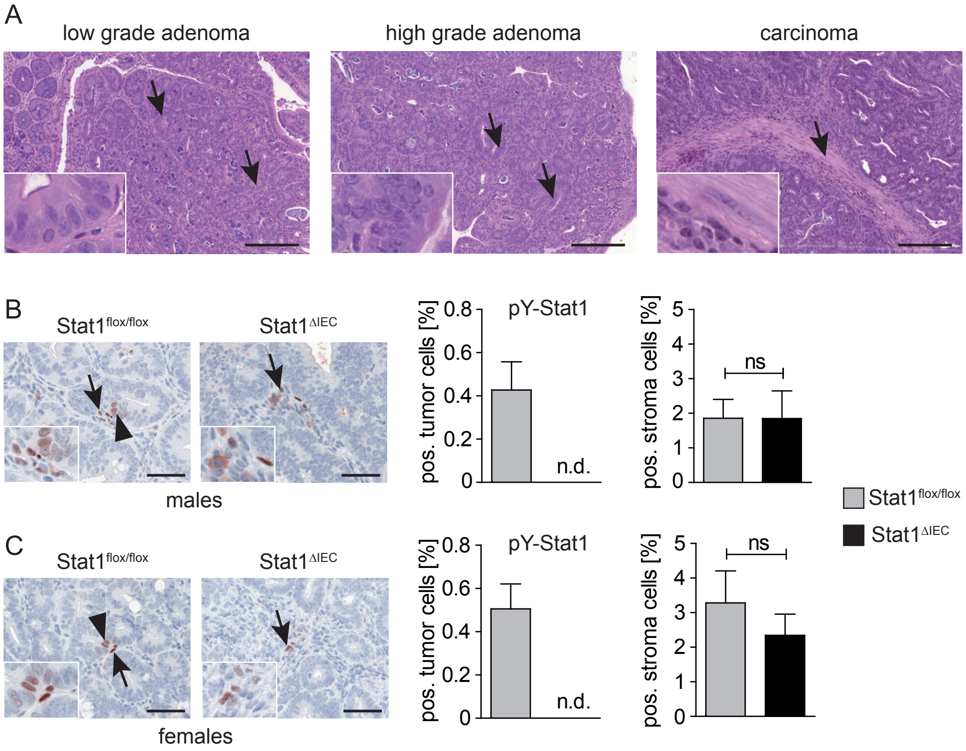

Supplemental Figure 4

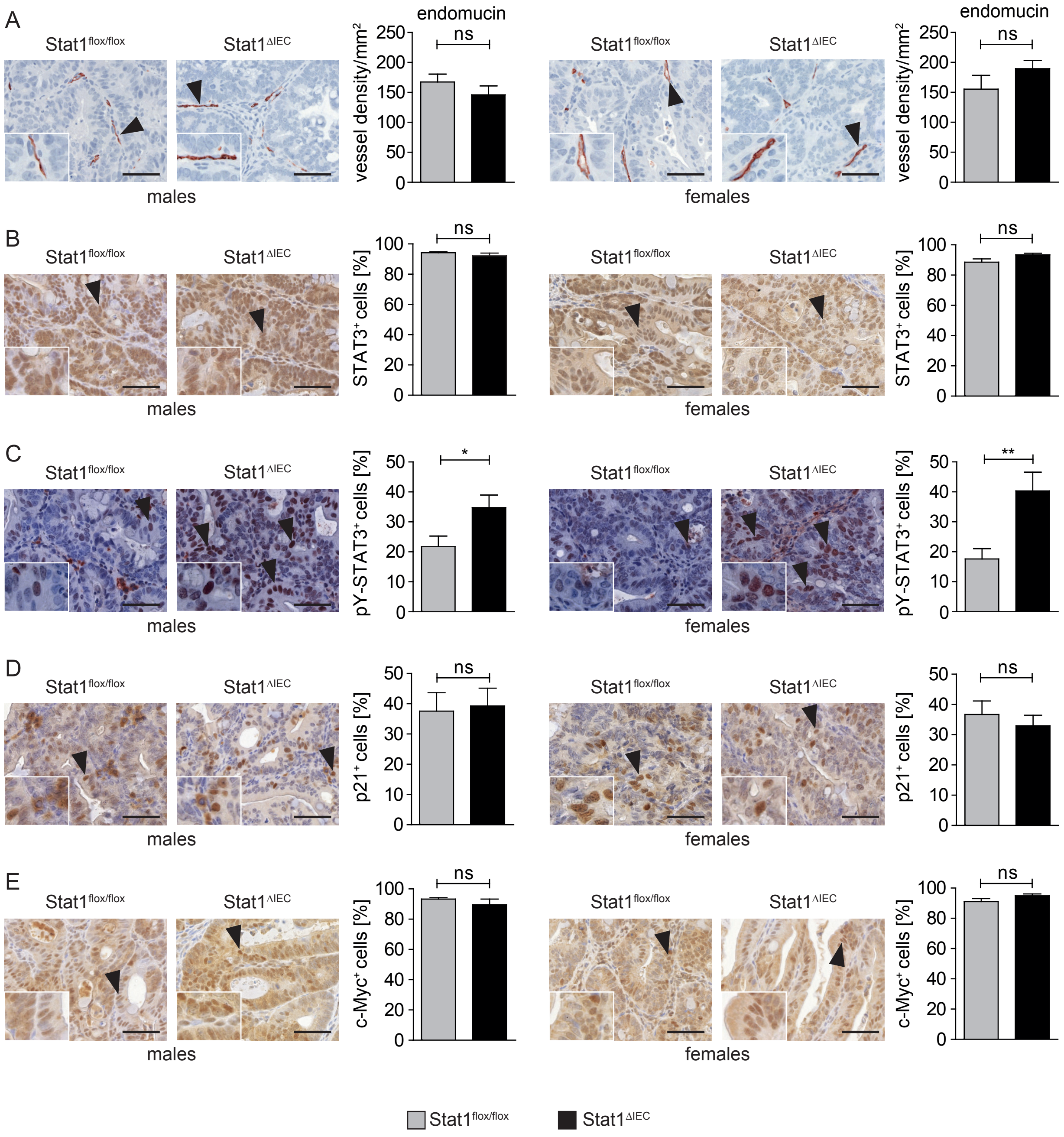

Supplemental Figure 5

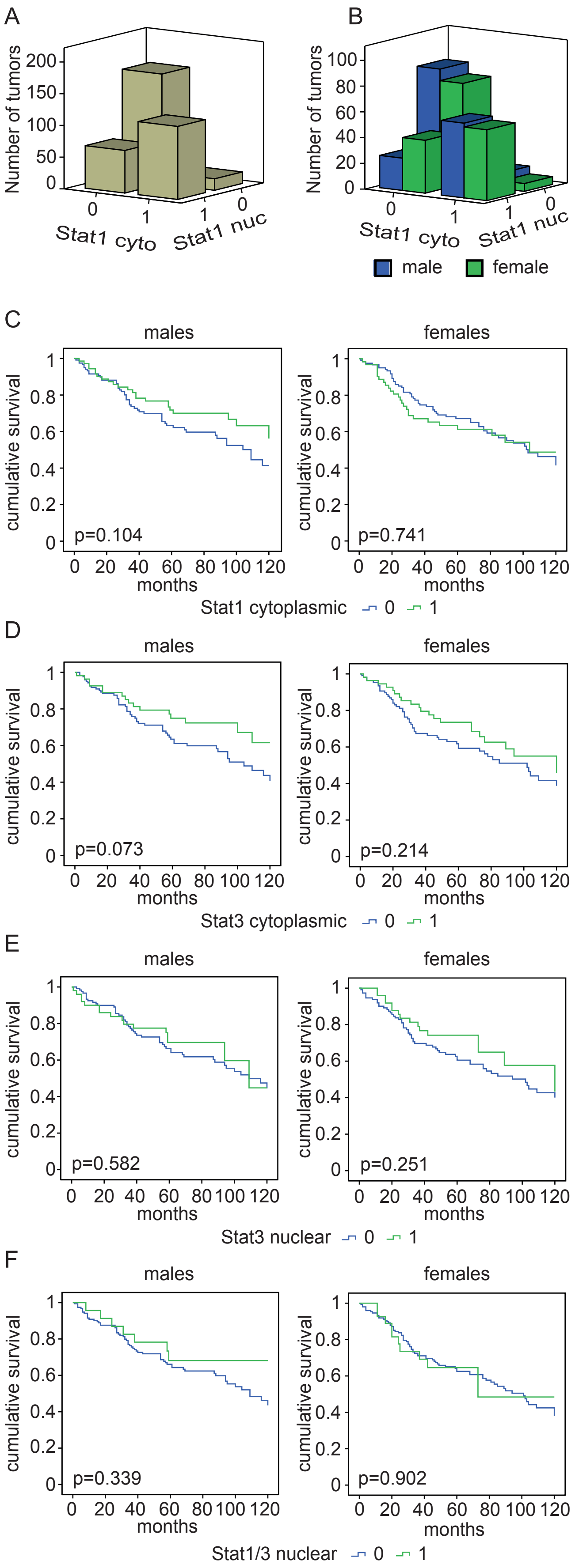

Supplemental Figure 6

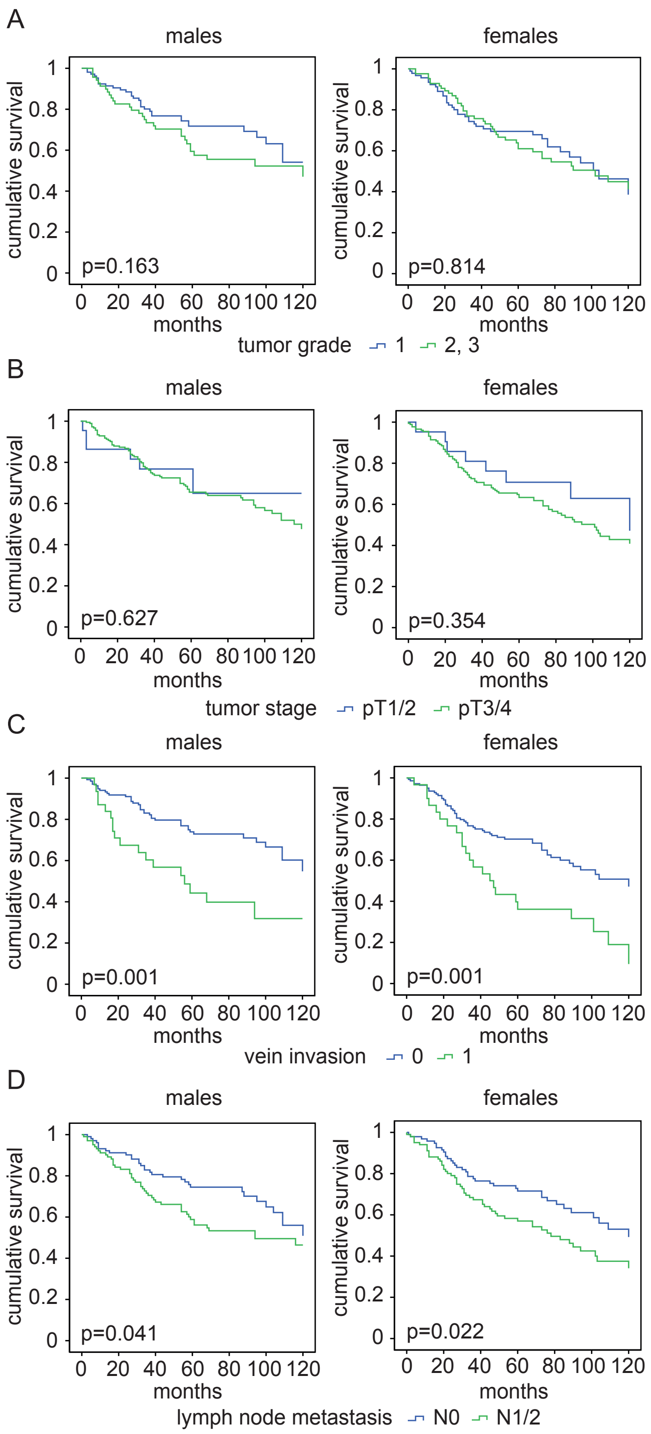

Supplemental Figure 7

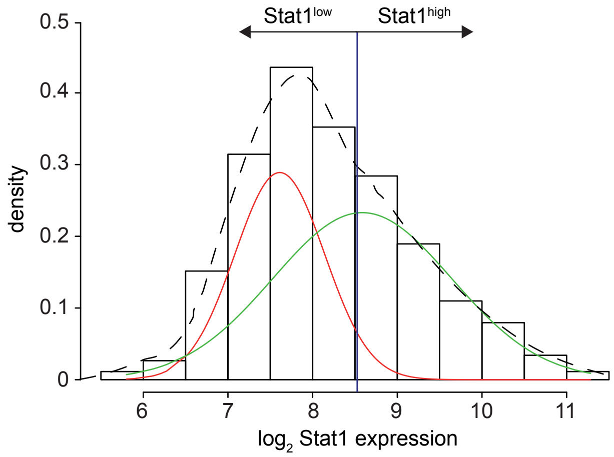

Supplemental Figure 8

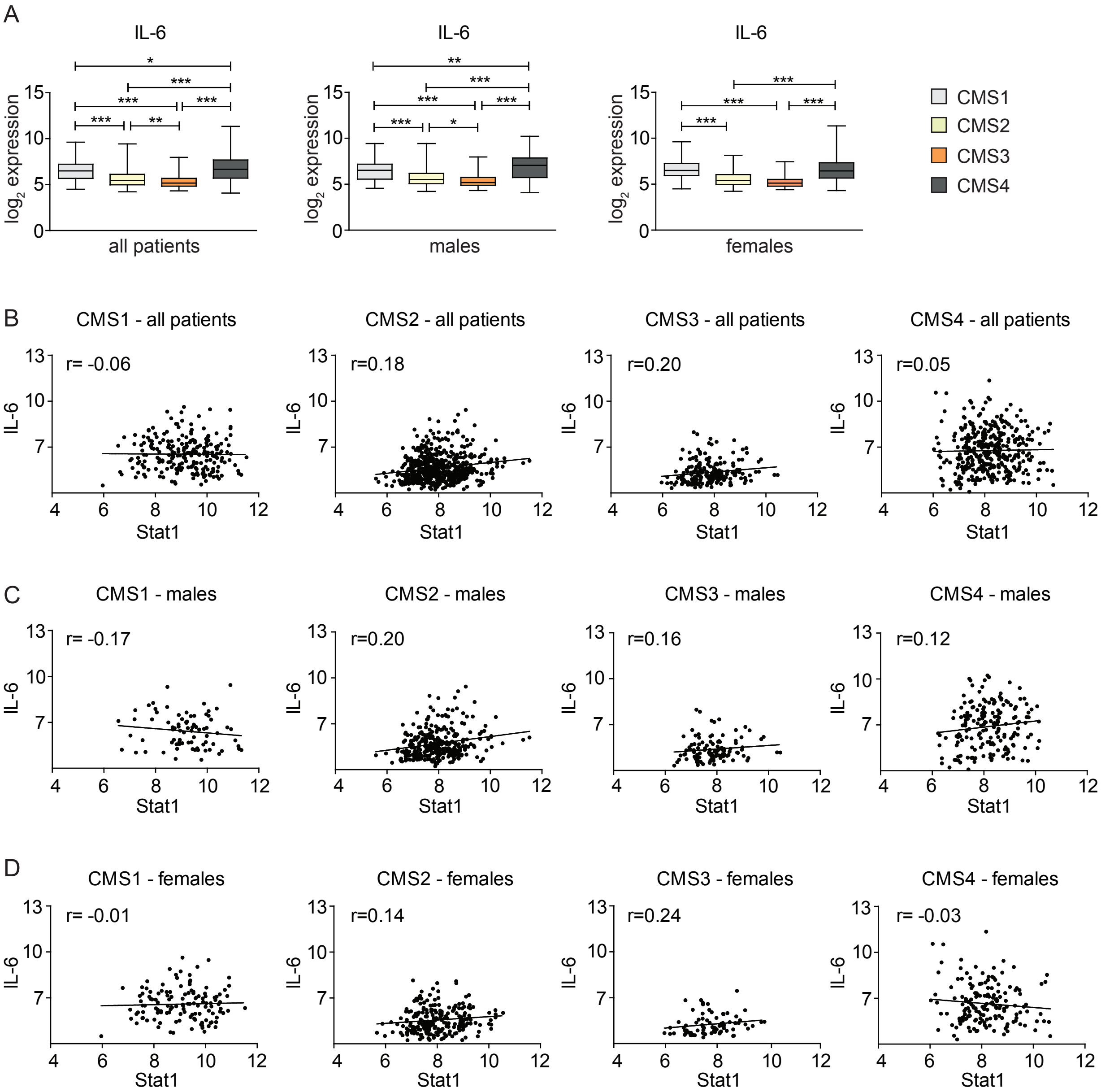

Supplemental Figure 9

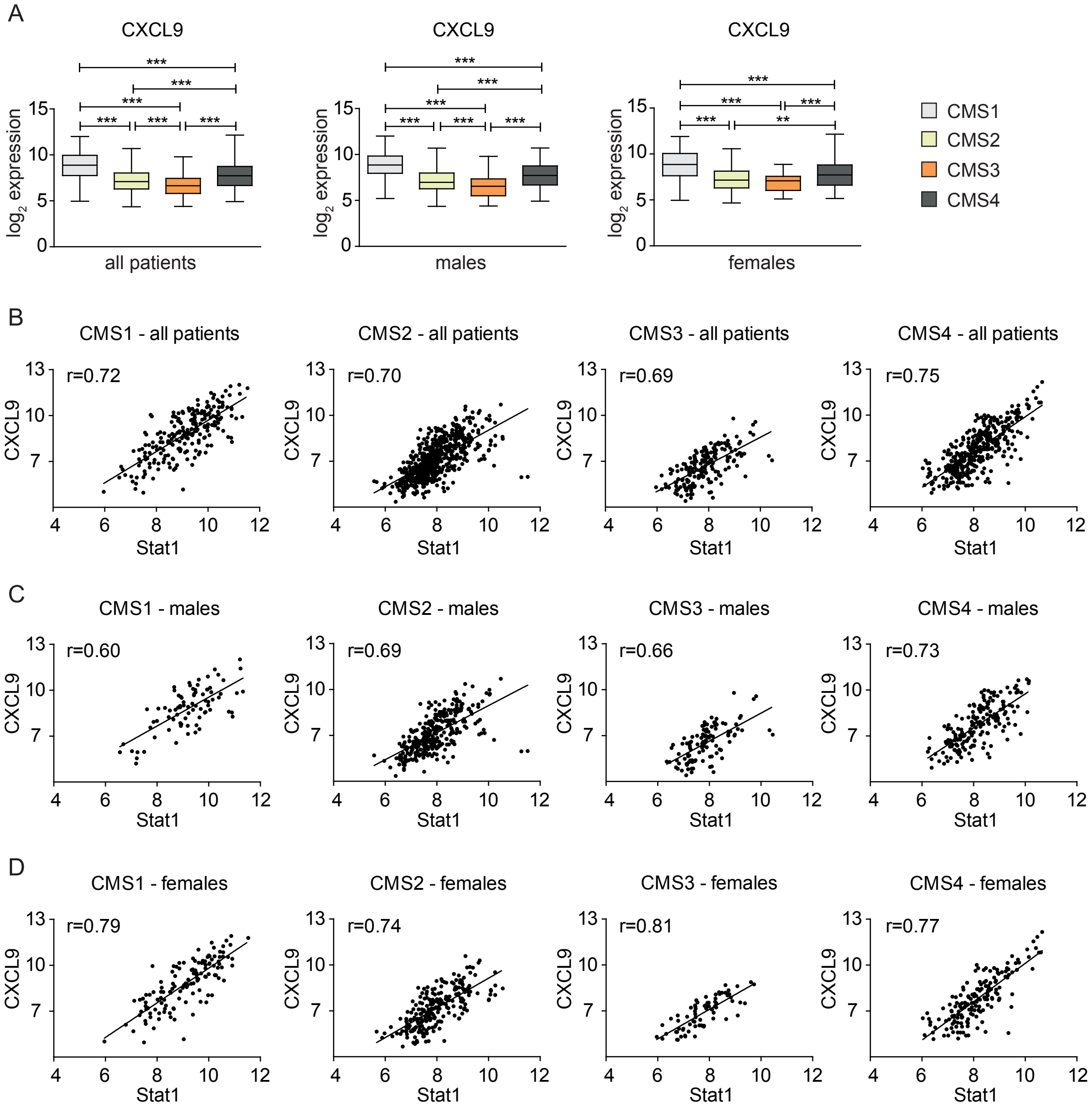

Supplemental Figure 10

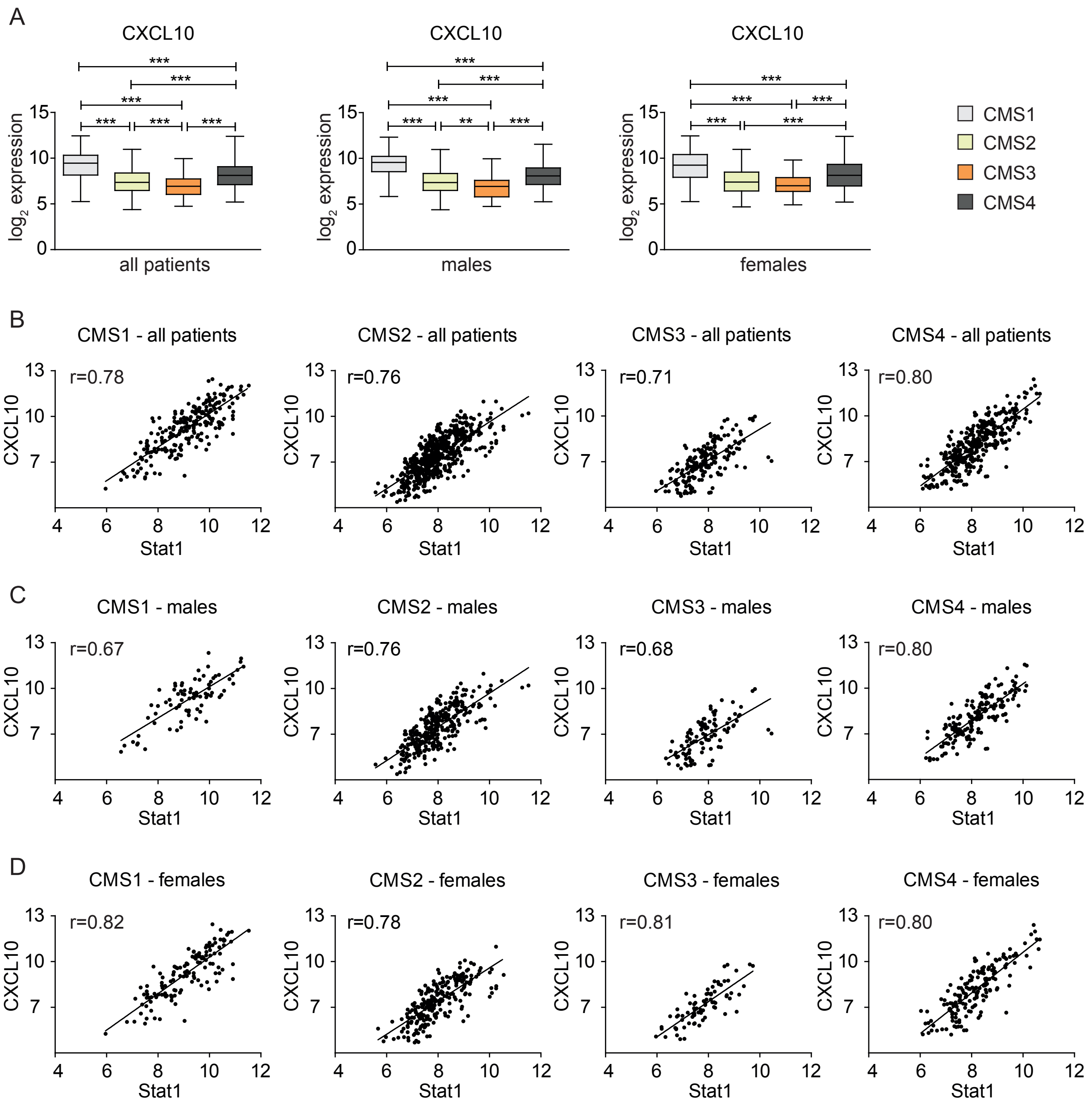

Supplemental Figure 11

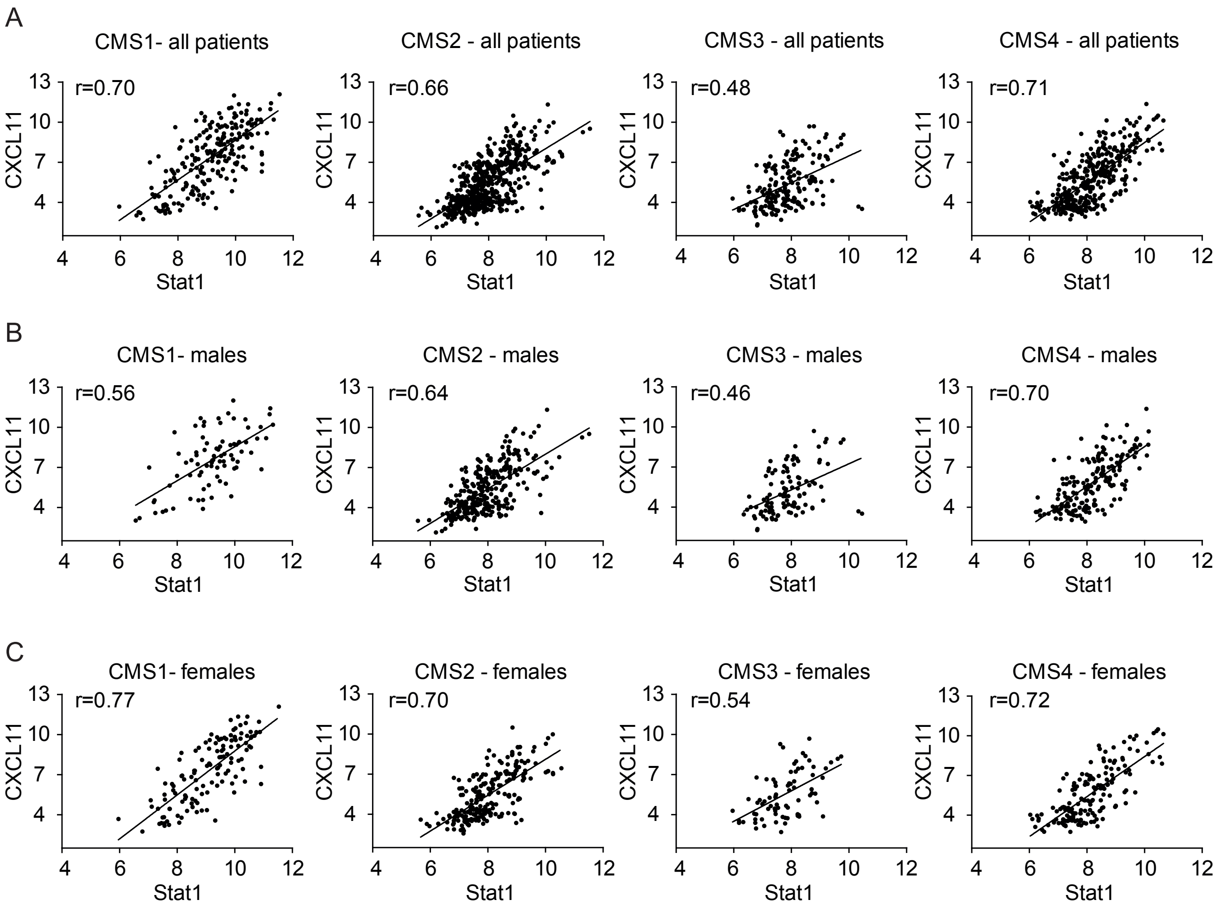

Supplemental Figure 12

Supplemental Table 1. No correlation of tumor cell-intrinsic nuclear STAT1 expression and vein invasion. Tumor cell-specific nuclear STAT1 expression and vein invasion status of 308 patients (149 males and 159 females) were cross-tabulated. X^2-test revealed no significant correlation between the two factors in either of the sexes.

sex	STAT1	vein invasion			p value
		negative	positive	total	
male	negative	64	16	80	0.684
	negative (%)	80%	20%	100%	
	positive	57	12	69	
	positive (%)	82.6%	17.4%	100%	
	total	121	28	149	
	total (%)	81.2%	18.8%	100%	
female	negative	61	14	75	0.456
	negative (%)	81.3%	18.7%	100%	
	positive	72	12	84	
	positive (%)	85.7%	14.3%	100%	
	total	133	26	159	
	total (%)	83.6%	16.4%	100%	
all	negative	125	30	155	0.379
	negative (%)	80.6%	19.4%	100%	
	positive	129	24	153	
	positive (%)	84.3%	15.7%	100%	
	total	254	54	308	
	total (%)	82.5%	17.5%	100%	

Supplemental Table 2. No correlation of tumor cell-intrinsic nuclear STAT1 expression and lymph node metastasis. Tumor cells specific nuclear STAT1 expression and lymph node metastasis status of 366 patients (183 males and 183 females) were cross-tabulated. X^2-test revealed no significant correlation between the two factors in either of the sexes.

sex	STAT1	pN			p value
		pN0	pN1/2	total	
male	negative	49	51	100	0.957
	negative (%)	49%	51%	100%	
	positive	41	42	83	
	positive (%)	49.4%	50.6%	100%	
	total	90	93	183	
	total (%)	49.2%	50.8%	100%	
female	negative	44	43	87	0.827
	negative (%)	50.6%	49.4%	100%	
	positive	47	49	96	
	positive (%)	49%	51%	100%	
	total	91	92	183	
	total (%)	49.7%	50.3%	100%	
all	negative	93	94	187	0.913
	negative (%)	49.7%	50.3%	100%	
	positive	88	91	179	
	positive (%)	49.2%	50.8%	100%	
	total	181	185	366	
	total (%)	49.5%	50.5%	100%	
